# Genomic and transcriptomic analyses support a silk gland origin of spider venom glands

**DOI:** 10.1186/s12915-023-01581-7

**Published:** 2023-04-13

**Authors:** Bingyue Zhu, Pengyu Jin, Yiming Zhang, Yunxiao Shen, Wei Wang, Shuqiang Li

**Affiliations:** 1 Key Laboratory of Zoological Systematics and Evolution, Institute of Zoology, Chinese Academy of Sciences, Beijing, 100101 China; 2 University of Chinese Academy of Sciences, Beijing, 101408 China; 3 Key Laboratory of Ecology and Environmental Protection of Rare and Endangered Animals and Plants, Ministry of Education, Guangxi Normal University, Guilin, 541004 China

**Keywords:** Comparative transcriptomics, Genomics, Gene co-expression networks, Gene selection pressure, Adaptive traits

## Abstract

**Background:**

Spiders comprise a hyperdiverse lineage of predators with venom systems, yet the origin of functionally novel spider venom glands remains unclear. Previous studies have hypothesized that spider venom glands originated from salivary glands or evolved from silk-producing glands present in early chelicerates. However, there is insufficient molecular evidence to indicate similarity among them. Here, we provide comparative analyses of genome and transcriptome data from various lineages of spiders and other arthropods to advance our understanding of spider venom gland evolution.

**Results:**

We generated a chromosome-level genome assembly of a model spider species, the common house spider (*Parasteatoda tepidariorum*). Module preservation, GO semantic similarity, and differentially upregulated gene similarity analyses demonstrated a lower similarity in gene expressions between the venom glands and salivary glands compared to the silk glands, which questions the validity of the salivary gland origin hypothesis but unexpectedly prefers to support the ancestral silk gland origin hypothesis. The conserved core network in the venom and silk glands was mainly correlated with transcription regulation, protein modification, transport, and signal transduction pathways. At the genetic level, we found that many genes in the venom gland-specific transcription modules show positive selection and upregulated expressions, suggesting that genetic variation plays an important role in the evolution of venom glands.

**Conclusions:**

This research implies the unique origin and evolutionary path of spider venom glands and provides a basis for understanding the diverse molecular characteristics of venom systems.

**Supplementary Information:**

The online version contains supplementary material available at 10.1186/s12915-023-01581-7.

## Background

The evolution of venom has long fascinated biologists, especially the complex venom systems that have evolved independently in more than 100 metazoan lineages to facilitate prey capture and defense [1, 2]. Spiders comprise a hyperdiverse lineage of predators with venom systems [3]. Most venom studies have concentrated on the recruitment and evolution of ecologically and/or pharmacologically important toxin genes, and they have shown that massive gene duplication, horizontal (lateral) transfer, and alternative splicing have given rise to diversity and different expression patterns in spider venom glands [4, 5]. Despite insights into the evolution and diversification of spider venom toxins [6], the origin and evolution of these functionally novel organs remain poorly understood [7].

Previous reviews have highlighted that spider venom systems are derived from the salivary glands or evolved from the silk-producing glands present in early chelicerates [8, 9]. For the former hypothesis, many digestive proteases that are also produced by spider venom glands cause tissue destruction and facilitate toxin penetration or are involved in the initial extra-oral digestion process of the prey [10–12]. Spider venom systems were thus assumed to be derived from modified salivary glands [9, 13], similar to snakes [14] and a handful of insectivorous mammals [2]. For instance, the deep homology of snake oral venom systems and mammalian salivary glands was examined at the level of regulatory architectures by focusing on gene co-expression networks [15].

For the latter hypothesis, the spinning glands in the chelicerae of some sea spider larvae may have been repurposed as spider venom glands [8, 9, 16]. Additionally, unexpected similarities between the biological features of spider venom glands and spider silk glands also support the connection between them. For example, they both develop from the ectoderm [17]; spidroins are expressed in venom gland RNA-Seq [18], and spitting spiders spit toxic silk droplets toward their prey by producing silk proteins in their venom glands [19]. However, it is still unclear which hypothesis is more reliable. To our knowledge, there is no evidence for proving homology between spider venom glands and the salivary glands or silk glands of other arthropods. Whether spider venom systems share a commonly conserved gene regulatory foundation with the salivary glands or silk glands across arthropods is an open question.

To test two alternative hypotheses, we first integrated the genomes and RNA-Seq datasets from various lineages of spiders, scorpions, ticks, mites, centipedes, and insects (Additional file 1: Table S1). We updated the genome of a model spider, the common house spider (*Parasteatoda tepidariorum*), to chromosome-level assembly for a better comparative analysis. Second, we analyzed and compared the gene expression patterns between spider venom glands and other tissues. To explore the specificity of expression in spider venom glands, we obtained the upregulated genes and specific transcription modules of the venom glands, as well as examined the influence of gene duplication and selection pressure on the expression variations. Our results contribute to understanding the origin and evolution of spider venom glands, which provide an important basis for further research on spider venom systems.

## Results

### Genome update of the common house spider

To better study the spider gene regulation model, high-quality genomes were needed for this analysis. We updated a chromosome-level common house spider genome by using 164.22 X Hi-C data and version 3.0 assembly (see the “Methods” section). Our assembly (~ 1.13 Gb) has a high continuity with the scaffold N50 value of 93.88 Mb (Additional file 2: Fig. S1a, b), which is more than 120 times better than that of the previous assembly [20]. The genome completeness score was 98.1% using the BUSCO of Arachnida (Additional file 2: Fig. S1c) [21], which was almost the same as the score of version 3.0 (98.0%). The genome assembly contained 12 pseudo-chromosomes (Additional file 2: Fig. S1b) [22]. For annotation, the gene model information of the previous version was aligned to the chromosome-level assembly. A total of 20,182 protein-coding genes were predicted, with 14.1% redundant genes reduced (Additional file 2: SI Text 1, Fig. S2) [20] and a BUSCO completeness of 97.4% (Additional file 2: Fig. S1c), indicating a high annotation accuracy.

### Co-expression network construction for spider venom glands

**Fig. 1:**
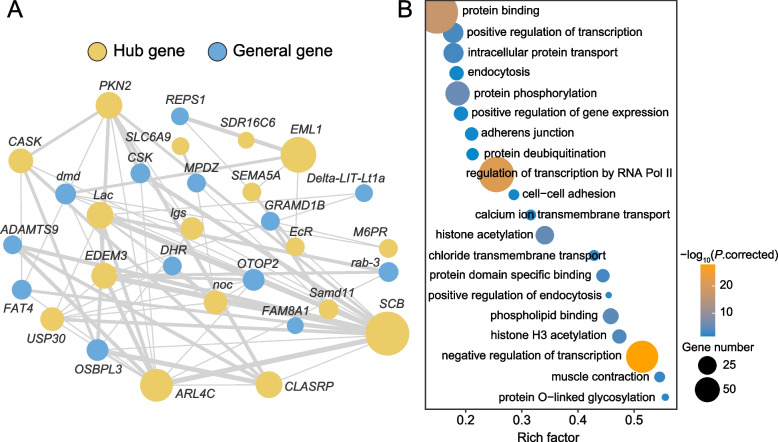
Core network module of the common house spider venom glands. **a** Cytoscape plot of the core network module. We termed the largest module that contained 2765 genes in the venom glands the core network, which has great functional relevance to the venom glands. Of these, 17 hub genes with the highest degrees of connection and their connections were visualized. Line thicknesses indicate the interaction strengths, and circle sizes represent the connection degrees. **b** GO enrichment of genes in the core network module. The complete enrichment results are shown in Additional file 1: Table S6

To clarify the gene association patterns in venom gland samples, we constructed a co-expression analysis network by using weighted gene co-expression network analysis (WGCNA) [23]. We generated 22 modules ranging in size from 102 to 2765 based on 18 venom gland RNA-Seq datasets (Additional file 1: Table S2; Additional file 2: Fig. S3). We found that most transcription factors (TFs, ~ 21.0%; Additional file 1: Table S3) and differentially upregulated genes for venom glands (DUGs, ~ 16.7%; Additional file 1: Table S4; Additional file 2: Fig. S4) were distributed in the largest turquoise module (module 1), implying that these genes are of great functional relevance in the venom glands. Therefore, we termed module 1 the core network (Fig. 1a). Unexpectedly, only a few previously identified toxin-coding genes [12] or genes detected in the venom proteome [24] were found in module 1, and many of them were assigned to the grey60 module (module 16; Additional file 1: Table S5), suggesting that the core network may have relatively weak regulation for toxins in the common house spider venom glands.

We checked the hub genes in module 1, which are integral to a network and have a high correlation in candidate modules [23]. The hub gene, *SCB*, had strong links with other highly expressed hub genes in venom glands (Fig. 1a). *SCB* is involved in animal organ development [25]. The toxin gene, *Delta-LIT-Lt1a*, had links with the hub genes *EDEM3* and *Lac*: the former accelerates the degradation of misfolded glycoproteins in the endoplasmic reticulum (ER) [26], and the latter regulates organ size by influencing cell length, indicating a role in cell adhesion [27]. We then defined the biological significance of the core network by Gene Ontology (GO) functional enrichment, which is mainly involved in transcription regulation, protein modification, transport, and signal transduction (Additional file 1: Table S6; Fig. 1b). These results suggested various protein secretory functions of venom glands.

### Ortholog expression patterns among species

We retained 1983 one-to-one orthologous genes from all ten arthropod species, including spiders, scorpions, ticks, mites, centipedes, and insects (Additional file 1: Table S1). Based on the orthologs, we created an expression matrix of log-transformed and quantile-normalized transcripts per million (TPM) values (see the “Methods” section). Multiple transcriptome samples (various glandular tissues, brains, ovaries, or fat bodies) were used for comparative transcriptomic analysis.

**Fig. 2:**
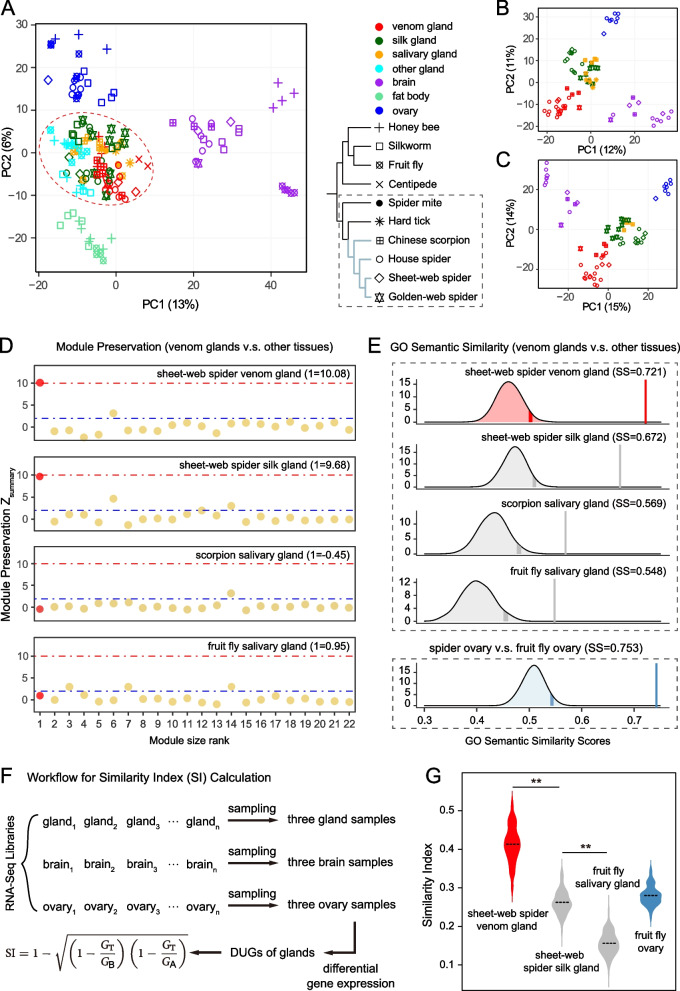
The expression patterns between the spider venom glands and other tissues across multiple species. **a** PCA using the expression levels of 1983 orthologs from ten species. The shapes and colors of the points represent species and tissues, respectively. The red dotted circles represent the clustering of glandular tissues. **b** PCA of the 2388 ortholog expression matrix across six species in the dotted box. **c** PCA of the 3952 ortholog expression matrix across four species, whose branches are shown in light blue. **d** Module preservation between the common house spider venom glands and other tissues. *Z*_summary_ > 10 implies strong preservation; *Z*_summary_ values between 2 and 10 indicate weak to moderate evidence of preservation; if *Z*_summary_ < 2, there is no evidence that the module is preserved. The red dot (module 1) represents the core network. **e** The observed pairwise semantic similarity (SS) scores and permutated ones between the common house spider venom glands and other tissues. Of these, the fifth density plot represents the high similarity between the spider ovary and fruit fly ovary (this value was used as a control in our analysis). The vertical lines show the observed pairwise SS values. The shades show 1000 permutated SS values with 95th and 90th percentiles labeled. **f** Workflow for similarity index (SI) calculations among the DUGs of glandular tissues. See the “Methods” section for the meaning of the equation. SI, similarity index; DUGs, differentially upregulated genes. **g** SI comparisons among the DUGs of the common house spider venom glands and glandular tissues. The comparison result between the spider ovary and fruit fly ovary is labeled in blue, showing high similarity (this value was also introduced as a control). The dotted line in the violin plot represents the median value. **FDR < 0.01 (Mann‒Whitney *U* test). See Additional file 2: Fig. S7–S10 for other comparison results

To obtain an overview of the ortholog expression patterns across species, we performed principal component analysis (PCA) by comparing a total of 154 RNA-Seq datasets (Additional file 1: Table S2). The PCA produced clear organ-wise segregation in the multispecies comparison across phylogenetically diverse lineages: the body tissues were separated, and glandular tissues that share basic secretion function showed similar expression profiles (Fig. 2a). In this step, the venom glands did not show clear similarity biases to the salivary glands or silk glands.

We expected that the fewer species included in our dataset, the higher the number of orthologous genes will be available for comparison, and the greater the possibility that each glandular tissue will be clustered separately. We next detected what happens to the clustering results between the venom glands and other glands if we reduce the number of species in our dataset. When PCA was performed using more orthologs based on six (spiders, scorpions, ticks, and mites) or four (only spiders and scorpions) species, the venom glands could be separated from the silk glands and salivary glands (Fig. 2b, c), which suggested specific venom gland expressions. These PCA cluster similarities were also supported by hierarchical clustering expression profile analyses and neighbor-joining expression tree constructions (Additional file 2: Figs. S5 and S6). However, exploration of ortholog expression patterns has not yet revealed the homology of the venom glands with the salivary glands or silk glands.

### Conservation comparison between spider venom glands and other tissues

To examine whether there are conserved molecular characteristics between the venom glands and other glandular tissues, we further performed a series of preservation and similarity analyses.

#### Module preservation

To test whether the modular characteristics of the venom gland-core network are preserved in other glandular tissues, we performed a module preservation analysis by estimating the conservative of gene densities and gene connections, which has proven to be feasible in conservatism comparisons [15]. The core network was preserved in the venom glands of another spider, with *Z*_summary_ > 10 (Fig. 2d), suggesting very high preservation. This result illustrated the similarity among arachnids rather than species specificity. Surprisingly, the *Z*_summary_ value was 9.68 in spider silk glands, indicating moderate preservation (Fig. 2d). In silkworm silk glands, the core network was weakly preserved (*Z*_summary_ = 3.43; Additional file 2: Fig. S7). In contrast, in the salivary glands of the scorpion and fruit fly, the core network had *Z*_summary_ < 2, displaying no preservation (Fig. 2d). Similar situations were also found in the salivary glands of mites (Additional file 2: Fig. S7).

#### Comparison of highly expressed gene functional enrichment

To inspect the credibility of similarities, we compared the highly expressed genes of glandular tissues using enriched biological processes (BPs) represented by GO terms (Additional file 1: Table S7). The pairwise semantic similarity (SS) of the GO annotations between two spider venom glands was 0.721 (Fig. 2e), signifying high similarity. The SS values between the venom glands and salivary glands (scorpion and fruit fly) were 0.569 and 0.548, respectively (Fig. 2e), which were significantly lower than the SS between venom and silk glands (0.672). Additionally, the similarity between venom glands and mite salivary glands was not low (0.639; Additional file 2: Fig. S8), which may be because these mite salivary gland samples contained other head tissues (e.g., silk glands in mites; Additional file 2: Fig. S9) [28], and spider mite salivary glands might secrete toxins [29].

Comparisons of the ovary or brain tissues between different taxa showed relatively high similarities (Fig. 2e; Additional file 2: Fig. S8), which suggests that the cross-species comparisons of homologous tissues were reliable, and the small similarities between the venom and salivary glands were likely not caused by cross-species analyses. All of the observed SS values were greater than the 95th percentile value of the permutations, revealing that they were statistically significant. These results implied no high degree of similarity between the venom glands and salivary glands.

#### Similarity of DUGs among glandular tissues

To further measure the expression conservation for the venom glands and other glandular tissues, we estimated the similarities among the DUGs of each tissue using a similarity index (SI; Fig. 2f) [30]. The median SI value of DUGs between the two spider venom glands was 0.42. However, between the venom and salivary glands, the median SI values were only 0.21 and 0.16 (Fig. 2g; Additional file 2: Fig. S10a), which were similar to the results between the venom glands and other glandular tissues but lower than those between the venom glands and silk glands. To account for shifts in paralog expressions in glandular tissues among species, we calculated corrected SI values, whose trends are similar to those of the SI comparison (Additional file 2: Fig. S10a), further improving the credibility of our analysis. We found that the DUGs of the ovaries or brains between different species also displayed relatively high similarities (Fig. 2g; Additional file 2: Fig. S10b, c), overcoming the differences among taxa and further suggesting that cross-species comparisons are not the reason for the low similarity between the venom glands and salivary glands. Our estimations suggested relatively low transcription similarities among the venom glands and salivary glands.

### Gene duplication may drive the differentiation of venom and silk glands in spiders

**Fig. 3:**
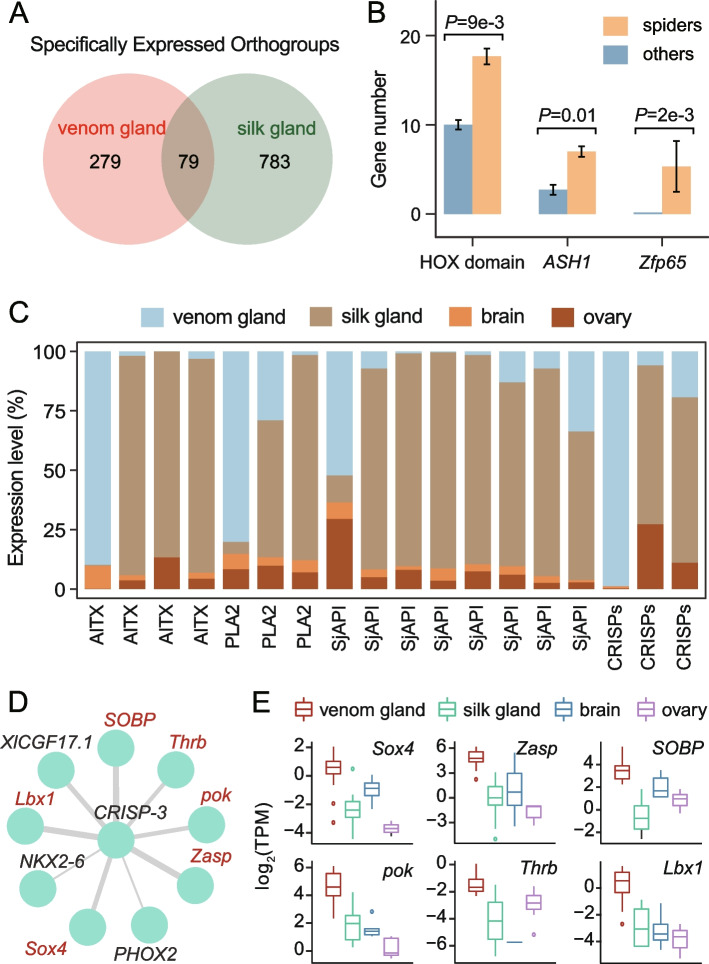
Expression differentiation of gene families in the common house spider venom glands and silk glands. Our results clarified that expression divergences of gene duplication events (including TFs) are common in venom and silk glands. **a** Venn diagram of specifically expressed orthogroups in spider venom and silk glands. The orthogroup in which a specifically expressed gene is located is considered to be specifically expressed. A total of 358 and 862 gene orthogroups were specifically expressed in the venom and silk glands, respectively, with 79 orthogroups shared between both tissue types. **b** TF orthogroups shared between the venom and silk glands showing expansions in spiders. *P*-values indicate significant differences in gene numbers (Mann‒Whitney *U* test). **c** Relative expression abundances of specifically expressed toxin paralogs in the venom and silk glands. **d** Nine TFs showing high module membership with the toxin gene *CRISP-3*. These genes were specifically expressed in spider venom glands. Line thicknesses indicate the interaction strengths. Six genes labeled red represent the upregulation in the venom glands.** e** Six upregulated TFs associated with the toxin gene *CRISP-3*. Boxes show the range, upper and lower quartiles, and median. HOX domain, TFs containing the HOX domain; *ASH1*, achaete-scute homolog 1; *Zfp65*, zinc finger 65; AITX, kappaPI-actitoxin-Avd3c; PLA2, phospholipase A2; SjAPI, venom peptide SjAPI; CRISPs, cysteine-rich secretory proteins; *CRISP-3*, cysteine-rich secretory protein 3; *Lbx1*, transcription factor LBX1; *NKX2-6*, homeobox protein Nkx-2.6; *PHOX2*, paired mesoderm homeobox protein 2; *pok*, ets DNA-binding protein pokkuri; *SOBP*, sine oculis-binding protein homolog; *Sox4*, SRY-Box transcription factor 4; *Thrb*, thyroid hormone receptor beta-A; *XlCGF17.1*, gastrula zinc finger protein XlCGF17.1; *Zasp*, PDZ and LIM domain protein Zasp

Spider venom and silk glands reflect their conservative characteristics in terms of module preservation and expression similarities, implying lineage-specific constraints that are related to specific secretory functions. We tried to examine the link between gene expression and tissue differentiation. The specifically expressed genes in the tissues were defined as those that had *τ* indexes exceeding 0.8 and were expressed most strongly in these tissues [31]. By comparing the specific gene orthogroups of the venom glands and silk glands, we found that a total of 629 genes (358 orthogroups) were specifically expressed in the venom glands (Additional file 1: Table S8), of which 226 paralogs (79 orthogroups) were specifically expressed in the silk glands (Fig. 3a). A total of 13 TF orthogroups (~ 16.5%) were shared between the venom glands and silk glands, with three of them showing expansions in spiders (Fig. 3b). For example, the TF *ASH1* orthogroup showed ancient duplications in arachnids, with the distribution of paralogs concentrated on chromosome 10 (Additional file 1: Table S9; Additional file 2: Fig. S11). We then found that more than 700 paralogs of venom gene-associated modules showed tissue-specific expressions (Additional file 2: SI Text 2, Figs. S12 and S13), of which ~ 35.1% were silk gland-specific expressions (Additional file 2: Fig. S14). The paralogs of the coding genes for cysteine-rich secretory proteins (CRISPs), phospholipase A2 (PLA2), and other toxins were also specifically expressed in the silk glands (Fig. 3c). Notably, nine venom gland-specific TFs had strong interactions with the toxin gene, *CRISP-3* (Fig. 3d), six of which were differentially upregulated in the venom glands and expressed at low levels in the silk glands (Fig. 3e), showing possible differences in *CRISP-3* regulation in the two tissue types. Our results suggested that expression divergences of gene duplication events (including TFs) are common in the venom glands and silk glands, further implying that gene cooperative interactions may drive the evolution of the core regulatory complex, which in turn is accompanied by the formation of new organs or new cell types [32].

### Gene variations in specific transcription modules of the venom glands across spiders

**Fig. 4:**
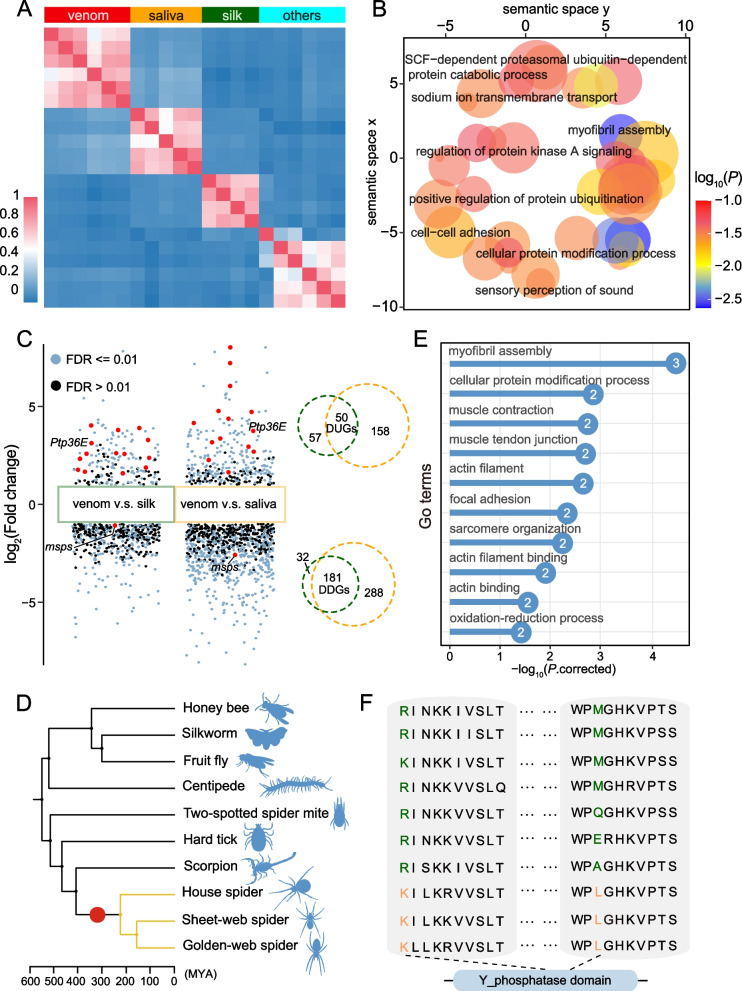
Gene variations in specific transcription modules of spider venom glands. **a** Heatmap of Spearman correlation coefficients between specific modules of glandular tissues. Venom gland-specific modules contain 166 orthologs found through the isa2 method. venom, venom gland; silk, silk gland; saliva, salivary gland; others, other glands, including the prothoracic, hypopharyngeal, and lymph glands of insects.** b** REVIGO clusters of the significantly enriched GO terms for venom gland-specific modules. Bubble sizes indicate the number of GO terms in each cluster, and the colors represent the corrected enrichment *P*-value on a log_10_ scale. Similar clusters plot closer to each other. **c** Differential expression analysis shows up- and downregulated genes in spider venom glands. The venom glands were compared with spider silk glands (green box and circle) and all salivary glands (orange box and circle). False discovery rates (FDR) ≤ 0.01 are indicated by blue dots, while FDRs > 0.01 are indicated by black dots. Red dots signify the positively selected genes at the ancestor branch of spiders, as well as the genes in specific transcription modules. Digits in the Venn diagrams represent differentially expressed gene numbers in each group. *Ptp36E*, protein tyrosine phosphatase 36E; *msps*, mini spindles; DUGs, differentially upregulated genes; DDGs, differentially downregulated genes. **d** Time tree of ten species across arthropods. Yellow branches indicate the spiders used in our study; the red dot signifies the spider ancestor. MYA, million years ago. **e** GO enrichment of fourteen positively selected genes at the ancestral branch of spiders. These genes were contained in the venom gland-specific transcription modules and were differentially upregulated in the venom glands. Digits in the circles indicate the gene numbers enriched in the terms. **f** Specific mutation of the gene *Ptp36E*. Two sites (orange) of this gene were positively selected at the spider ancestor branch. This gene was under relaxed selection at three spider branches

Although the expression profiles were relatively conserved (Fig. 2a), specific gene regulation differed among the venom glands and other glandular tissues. To test the specific expression patterns among spider venom glands, we identified the gene modules of glandular tissues based on the ortholog expression matrix from package isa2 [33]. We observed 100 modules in total (Additional file 2: Fig. S15). Most modules included samples of different glandular tissue types (Additional file 1: Table S10), further suggesting their deep homology. We found 55 modules including venom gland samples, and of these, six were spider venom gland-specific (Fig. 4a), which differed in sample compositions (9 to 17 samples) and numbers of orthologous genes (24 to 161 orthologs). A total of 166 orthologs represented the venom gland-specific modules (Additional file 1: Table S11), which contained 12 TFs (~ 7.23%). We screened the GO functional categories. The genes in the venom gland-specific modules were mainly related to protein modification, signal transduction, and muscle activity (Additional file 1: Table S12; Fig. 4b).

We explored the influences of the variations in specific transcription modules on the evolution of the venom glands. To clarify the variations in gene expression, we obtained the differential expressions of specific modules in spider venom glands by comparing them to silk and salivary gland transcriptome samples (Fig. 4c). A total of 40 genes in venom gland-specific modules showed differential expressions, of which 38 were DUGs (Additional file 1: Table S13), showing the contribution of high expressions to tissue specificity.

We next asked if there was evidence for variations in gene evolutionary rates in response to the evolution of new organs. We found that ~ 42.2% of the genes in specific modules were positively selected at the spider ancestor branch (Additional file 1: Table S11). A total of 14 DUGs were under positive selection in specific modules (Additional file 1: Table S14; Fig. 4d), which were mainly related to muscle activity and protein modification (Fig. 4e), suggesting a stronger function. In particular, one of the positively selected DUGs (*Ptp36E*) was under relaxed selection on the spider branches (*K*-value = 0.67 and *P*-value = 1e − 4; Fig. 4f), which is predicted to be involved in protein dephosphorylation [34], revealing the shift to functional conservation. Additionally, the specific gene, *msps*, which is associated with the mitotic process [35], showed positive selection and differential downregulation (Fig. 4c).

## Discussion

### Exploring the origin of spider venom glands

**Fig. 5:**
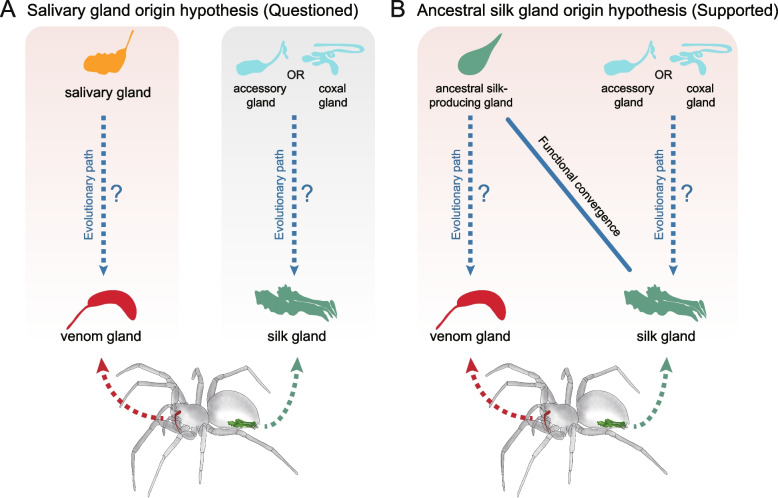
Hypotheses regarding the evolutionary derivation of spider venom glands. **a** Salivary gland origin hypothesis. We challenge this hypothesis based on the comparison results of module preservation, GO semantic similarity, and DUG similarity analyses (see Fig. 2). **b** Ancestral silk gland origin hypothesis. Our analyses prefer to support the concept that venom glands are likely derived from silk-producing glands present in early chelicerates. Previous assumptions were that spider silk glands evolved from accessory glands or were derived from the coxal glands [37, 38]. Modern spider silk glands may generate functional convergence with ancestral silk-producing glands, in turn resulting in high transcriptional similarities between spider venom glands and silk glands

Venom systems are one of the most successful adaptations in the animal kingdom [36]. In this study, we found that spider venom glands are less similar to salivary glands than to silk glands in transcriptional regulation, which calls into question the validity of the salivary gland origin hypothesis (Fig. 5a). Module preservation, GO semantic similarity and DUG similarity analyses revealed higher conservation between the venom glands and silk glands (Fig. 2d, e and g). Statistically, our analyses were unlikely to lead to these similarities. To reduce general errors, this research performed relatively stringent RNA-Seq sample retrieval, expression normalization, and multiple comparisons. Additionally, the PCA indicated the reliability of our ortholog dataset and revealed more significant differences among tissues than among species (Fig. 2a). Our results provide the first proof from a gene expression perspective that spider venom systems may not be modified from salivary glands (Fig. 5a).

Spider venom glands and silk glands share a similar molecular basis. For instance, the conserved core network of spider venom glands and silk glands is mainly related to transcription regulation, protein modification, transport, and signal transduction pathways (Fig. 1). The expression divergences of gene duplication events (including various TFs and toxin genes) are common in the venom glands and silk glands (Fig. 3), implying that gene duplication may precede or even drive the partitioning of ancient cells into distinct cell types. These results further reflected the close connection between the venom and silk systems [9].

Therefore, we prefer to support the silk gland origin hypothesis: spider venom glands were independently modified from ancestral silk-producing glands (Fig. 5b; Additional file 2: Fig. S16) [16, 28, 39, 40]. The classic view is that spider silk glands evolved from accessory glands or were derived from coxal glands [37, 38]. We hypothesized that spider silk glands may generate functional convergence with ancestral silk-producing glands, in turn resulting in the high transcriptional similarities between spider venom glands and silk glands. Either way, further research is needed to determine the evolutionary connection between spider venom glands and silk glands.

### Genetic differences among venom glands and other secretory systems

Although they share a gene regulatory foundation, secretory systems have phenotypic and functional differences in spiders and closely related groups. For example, the spinning glands of sea spider larvae produce web threads to bind the eggs together into a solid cocoon by combining the remnants of a cement substance [16], whereas spider venom glands produce a complex cocktail of diverse and selective natural products [41]. At the genetic level, these differences are reflected in our comparative results across arthropods. The genes in specific transcription modules of spider venom glands were mainly related to protein modification, signal transduction, and muscle activity (Fig. 4a, b). A considerable number of genes in specific modules displayed positive selection at the ancestral branch of spiders or upregulated expression in the venom glands compared to the silk and salivary glands (Additional file 1: Table S11 and S13; Fig. 4c–e), indicating that the adaptive change in protein secretion may correspond to the secretory system functional differentiation. The gene, *Ptp36E* (related to protein modification; Fig. 4f), in specific transcription modules was not only under positive selection in the common ancestor of spiders but was also with relaxed selection on the spider branches, indicating an improvement at the early time and conservation maintenance trends later for functional diversity of the proteome. These analyses suggest that changes in coding gene expression regulation and fitness effects may lead to the functional evolution and conservation of venom glands.

### Gene regulatory variations drive organ expressional changes

The evolution of TFs and gene regulatory networks affect gene expression, which in turn may explain many phenotypic differences among organs or species [32, 42]. We found that some TFs in venom gland-specific transcription modules showed gene duplications in spiders or were under positive selection (Additional file 1: Table S11). New or selected TFs may have functional differentiation, which combine the genes originally expressed in other tissues to form a specific regulatory complex of new organs [32, 43]. For example, some homologous TFs that show strong interactions with the toxin gene *CRISP-3* in spiders (Fig. 3d, e) were highly expressed in the fat bodies or prothoracic glands of the fruit fly (Additional file 2: Fig. S17), revealing the formation of specific functional modules and gene recruitment in different tissues across species. Furthermore, non-coding regulatory changes, such as promoters, enhancers, and other elements of toxin-coding genes in *Nematostella* [44], can also drive expressional divergences [45]. Further study with better annotation of non-coding regions could help illustrate the role of non-coding regulation on the expressional changes in different tissues.

Finally, PCA, hierarchical clustering of Spearman’s correlation coefficients, and expression tree construction (Fig. 2a; Additional file 2: Figs. S5 and S6) also indicated deep homology for the secretion feature of glandular tissues in spiders, scorpions, ticks, mites, centipedes, and insects beginning ~ 550 million years ago [46]. Thus, we cannot deny the possibility that spider venom glands may be modified from other exocrine glands or cell types based on their conserved secretory characteristics. In addition, it is hard to account for the effect of phylogenetic signals on the similarities between spider venom and silk glands that we observed, and we did not include other silk glands of chelicerates in our study, which was limited by difficulties such as RNA-Seq sampling for sea spider larvae. In the future, thorough studies are needed to verify the origin and evolution of spider venom glands, including but not limited to cell type analyses through comparative single-cell transcriptomics and investigation of expression regulation (variations) by epigenetic research.

## Conclusions

In conclusion, by investigating spiders and closely related groups of arthropods, comparative transcriptomics and genomics expanded our current understanding of the expression regulation of spider venom glands and other glandular tissues. We clarified the lower conservation and homology between the venom glands and salivary glands compared to the silk glands in the gene co-expression network, functional enrichment, and differential expression, as well as challenged the hypothesis that spider venom systems evolved from the salivary glands. We also revealed evolutionary shifts in spider venom glands through genetic analyses, which corresponded to protein modification, signal transduction, and muscle activity. Transcriptome similarity analyses suggested that spider venom glands and other glandular tissues across arthropods shared deep homology, tracing the origin of a conservative secretion feature to ~ 550 million years ago. Our findings propose ancient and conserved gene regulation in spider venom glands, which may facilitate research on the diversification and evolution of injection organs or venom gland cells.

## Methods

### Dataset construction

#### Species selection

To perform comparison analyses, we selected species across arthropods with relatively high-quality genomes, including the common house spider (*Parasteatoda tepidariorum*) [20], sheet-web spider (*Hylyphantes graminicola*) [12], batik golden web spider (*Trichonephila antipodiana*) [47], Chinese scorpion (*Mesobuthus martensii*) [48], hard tick (*Hyalomma asiaticum*) [49], two-spotted spider mite (*Tetranychus urticae*) [50], centipede (*Strigamia maritima*) [51], fruit fly (*Drosophila melanogaster*) [52], domestic silkworm (*Bombyx mori*) [53], and honey bee (*Apis mellifera*) [54]. Relatively more transcriptome data is available in public databases for most of the selected species. The coding sequences were downloaded from public databases (Additional file 1: Table S1). The longest transcript was retained when multiple transcripts were annotated to the gene.

#### Transcriptome data retrieval and sequencing

Transcriptome samples of multiple taxa tissues were downloaded from the NCBI Sequence Read Archive (SRA) database by pfastq-dump v0.1.6 (https://github.com/inutano/pfastq-dump), including the venom glands, salivary glands, silk glands, other glandular tissues, fat bodies, ovaries, and brains (Additional file 1: Table S2). Only data generated from healthy tissues were used. Where possible, at least three paired-end (PE) libraries for each tissue from each species were chosen, with bases between 6 and 8 Gb. For a larger RNA-Seq sample, we sampled length bases and retained 6–8 Gb using seqkit v2.1.0 [55] to reduce the impact of sample size differences on the following analyses. To remove low-quality reads and adapters, the downloaded transcriptomes were filtered through FASTP v0.20.1 [56]. We also utilized nine RNA-Seq samples of a sheet-web spider that were obtained in our previous study [12].

To supplement the sufficient expression data, we sequenced 67 transcriptomes, including the venom glands, silk glands, ovaries, and brains from the common house spider, as well as the venom glands, salivary glands, and brains from the Chinese scorpion (Additional file 1: Table S2). The common house spiders were collected in a non-motor vehicle garage of the Institute of Zoology, Chinese Academy of Sciences (IZCAS). Scorpions were collected from the Funiu Mountains, Xichuan County, Henan province, China. Total RNA was extracted using the RNAsimple Total RNA kit (TIANGEN, Beijing, China). PE 150 bp libraries were prepared and sequenced on an Illumina NovaSeq 6000 platform with an insert size of 250–300 bp. Raw sequences were filtered using FASTP, with ~ 6 Gb for each sample.

#### Genome sequencing, assembly, and annotation

The new genome assembly of the common house spider (*Parasteatoda tepidariorum*) (version 4.0) was obtained according to the previous genome assembly (NCBI genome ID: GCA_000365465.3), which was further scaffolded by Hi-C libraries. We constructed two Hi-C libraries using over 0.5 g of egg samples at Novogene (Beijing, China). Hi-C sequencing was conducted on an Illumina NovaSeq 6000 platform with PE 150 bp. A total of 201.99 Gb of clean data were obtained. Hi-C data were mapped to the genome by Juicer v1.6 [57]. The chromosome constructions were executed with 3D-DNA v180922 [58]. We performed the correction with Juicebox Assembly Tools v1.11.08 [59]. We attained a new annotation by using GMAP (2020–04-08) [60] to map annotation versions 2.0 and 3.0 (GCA_000365465.2 and GCA_000365465.3) of *P. tepidariorum* [20] to chromosome-level assembly. Genome completeness assessments were performed by applying the BUSCO v5.2.2 pipeline [21], searching the Arachnida (odb10; 2934 genes) dataset.

In addition, repeat sequences were identified using RepeatModeler v2.0.2 [61] and RepeatMasker v4.1.2-p1 [62]. Non-coding RNAs were also annotated. tRNAs were predicted using tRNAscan-SE v2.0.9 [63] with eukaryote parameters. Other non-coding RNAs were detected by searching against the Rfam database with Infernal cmscan v1.1.4 [64, 65].

#### Ortholog identification

One-to-one ortholog identification among ten species was performed using the reciprocal best-hit (RBH) method [66] by BLASTP in BLAST v2.10.0 + (*E*-value < 1e − 10) [67]. *Drosophila melanogaster* was used as an anchor species. We obtained 2110 raw orthologous genes for all ten species, 2504 genes for six species in arachnids, and 4031 genes for three spiders and scorpions. Nucleotide sequence alignments were obtained using MAFFT v7.455 with the G-INS-i model [68]. Poor alignments were trimmed through trimAL v1.4.rev15 (-gt 1 -st 0.001) [69]. Nucleotide sequences shorter than 50 bp were discarded. Sequence similarity and length consistency can reduce the impact on comparative transcriptomics analysis.

#### Ortholog expression matrix

We obtained ortholog expression matrices that had high-confidence alignments across multiple taxa based on one-to-one orthologs. We calculated gene expression values by mapping all RNA reads (Additional file 1: Table S2) back to the trimmed orthologs of each species using RSEM v1.3.1 [70], with the exception of *Trichonephila clavipes*, which was mapped to *T. antipodiana*, and *Rhipicephalus evertsi*, which was mapped to *Hyalomma asiaticum*. To allow comparisons of these data among biological replicates, tissues, and species, we normalized the gene expression data by transforming transcripts per million (TPM) scores to log_2_(*N* + 1) values. The transformed data were then quantile normalized among samples by the R package limma v3.40.6 [71]. We next removed the batch effects with the ComBat function in the R package sva v3.32.1 [72] by using the method from Barua and Mikheyev [15].

#### Reference genome-based RNA-Seq expression

To attain the complete expression information of the transcriptome samples, we also aligned all RNA-Seq data of each species to their own genomes using HISAT2 v2.1.0 [73], with the exception of *Trichonephila clavipes*, *Rhipicephalus evertsi*, and *Strigamia maritima*. The read counts of each gene were counted in the HTSeq framework v0.11.2 [74]. The gene counts of all samples were then normalized to TPM values based on the total reads per sample and effective gene lengths. A total of seven TPM matrices were obtained.

### Transcriptome similarity analysis

#### Sample cluster analysis

For transcriptome similarity comparisons, normalized ortholog expression matrices were used. We randomly used a subset of venom and silk gland data of the common house spider because of the relatively large number of samples. Principal component analysis (PCA) was carried out with the plotPCA function in the DESeq2 package v1.24.0 [75]. Spearman’s correlation coefficients between all pairs of samples were calculated in R v3.6.1 [76]. We executed hierarchical clustering of Spearman’s correlation coefficients by the “ward.D” agglomerative method and correlation distance. Heatmaps were generated in the R package pheatmap v1.0.12 (https://CRAN.R-project.org/package=pheatmap).

#### Gene expression phylogenies

Expression trees for all tissues were constructed with the neighbor-joining approach using functions in the ape package v5.6.2 [77] based on the pairwise distance matrices between samples. We calculated the pairwise distances from the 1-Spearman’s correlation coefficients.

### Weighted gene co-expression network analysis (WGCNA) using venom glands

The co-expression networks of the venom glands from the common house spider were constructed using the R package WGCNA v1.69 [23]. The input data consisted of the reference genome-based TPM expression matrix, with 18 venom gland transcriptome samples used, of which low-expressed genes were filtered (average TPM < 0.05). We attained an approximate scale-free topology by selecting a soft threshold of 9 based on results from the “pickSoftThreshold” function in the WGCNA package (Additional file 2: Fig. S3a). A height cutoff of 0.25 and a minimum module size of 30 were applied to merge very similar expression profiles with 22 modules obtained. The hub genes in the co-expression network were identified based on module membership correlations (more than 0.8) and significances between gene and trait (absolute value > 0.2). The network plots were generated by Cytoscape v.3.8.2 [78].

The “ModulePreservation” function in WGCNA was used to calculate module preservation pairwise statistics between the given reference set and all other test sets. For each reference-test pair, the function only uses genes that are common between the reference and test set [79]. We employed *Z*_summary_, which is a composite statistic that combines statistical summaries of network density and connectivity, to estimate the preservation of network characteristics between the reference and test sets. Generally, *Z*_summary_ > 10 implies strong preservation; a threshold of 2 > *Z*_summary_ < 10 indicates weak to moderate evidence of preservation; if *Z*_summary_ < 2, there is no evidence that a module is preserved [79].

### Differential expression analyses of spider venom glands

Differential gene expression analysis was carried out using the edgeR package v3.26.8 [80, 81] by comparing transcriptome samples from the venom glands, ovaries, brains, and silk glands in the common house spider. To examine sample clustering, we executed multidimensional scaling (MDS) using the “plotMDS” function. We then carried out differential gene expression analysis by an ANOVA-like test in edgeR, with a fold change > 2 and a 5% false discovery rate (FDR). In addition, to identify the differentially expressed genes (DEGs) of the venom glands across spiders, we also performed an ANOVA-like test by comparing them with spider silk glands and all salivary glands using the ortholog expression matrix of multiple species. Visualization of the differential gene expressions was performed with dot plots in ggplot2 v3.3.3 [82].

### Semantic similarity permutation

To evaluate the overall similarity of the functional enrichment patterns among glandular tissues, we calculated the pairwise semantic similarity (SS) values of the Gene Ontology (GO) terms and examined the SS values relative to random samples. Using a genome-based transcriptome expression matrix, we focused on the top 500 genes with the highest average expression for each glandular tissue. We did not include more genes because doing so means more similar GO functions and smaller differences in SS values across all groups. GO enrichment analyses were conducted using KOBAS 3.0 online tools (http://kobas.cbi.pku.edu.cn/; http://bioinfo.org/kobas) [83]. We identified the significantly enriched biological process terms (corrected *P*-values ≤ 0.05) using *Drosophila melanogaster* as the background reference species. We used the GoSemSim package v2.10.0 [84] and applied the BMA algorithm to calculate the individual SS values. To determine if the observed SS values significantly deviated from a random expectation, we sampled the same numbers of GO terms for test tissues from the pool of all 5046 biological process GO terms of the fruit fly 1000 times and calculated SS values. Values falling outside of the 95% confidence interval of the random samples were considered significant.

### Similarity index among the DUGs of glandular tissues

We compared the similarity index (SI) values among the DUGs of glandular tissues. Based on the ortholog expression matrix, we performed differential gene expression by comparing the glandular tissues with the ovaries and brains from the same species through edgeR (fold change > 2, *P*-value ≤ 0.05). The SIs were calculated with the following equation, where G_T_ is the number of shared DUGs, G_A_, and G_B_ are the total number of DUGs for tissues A and B, respectively, including both tissue-specific and shared DUGs [30]. To ensure that the results were robust, we sampled three libraries for each tissue type from each species and carried out differential gene expression analyses 100 times (Fig. 2f). For each pair of tissues, a total of 100 SI values were generated. The significances of the SI differences among groups were manually examined with the pairwise Mann‒Whitney *U* test in R. We obtained the FDR with the Benjamini and Hochberg method [85].

$$\mathrm{SI}=1-\sqrt{(1-\frac{{\mathrm{G}}_{\mathrm{T}}}{{\mathrm{G}}_{\mathrm{B}}})(1-\frac{{\mathrm{G}}_{\mathrm{T}}}{{\mathrm{G}}_{\mathrm{A}}})}$$

Considering comparisons of paralog expressions, we calculated the corrected SIs for glandular tissues by using reference genome-based RNA-Seq expression matrices, which treat paralogs as functionally equivalent. We first obtained the DUGs of glandular tissues using the same method as above. Next, we used OrthoFinder v 2.3.11 (-M msa -S diamond) to infer the gene families across ten taxa [86]. The gene families in which DUGs are located are considered to be differentially upregulated. The corrected SIs were then estimated based on the number of differentially upregulated gene families.

### Specific gene identification

We identified specific genes in different tissues of the common house spider by calculating tissue specificity indices *τ* [31]. *τ* is defined as follows:

$$\tau =\frac{{\sum }_{\mathrm{i}=1}^{n}(1-{\widehat{X}}_{i})}{n-1}; {\widehat{X}}_{i}=\frac{{X}_{i}}{\underset{1\le i\le n}{\mathrm{max}}({X}_{i})}$$

*X*_*i*_ is the expression of the gene in tissue *i*, and *n* is the total number of tissues. The *τ* value ranges from 0–1: a value close to 1 indicates tissue-specific expression, while a value close to 0 indicates ubiquitous expression. The specifically expressed genes were defined as those that had *τ* values > 0.8 and whose top three samples with the highest expressions were obtained from the same tissue type.

The orthogroup in which a specifically expressed gene is located is considered to be specifically expressed. We also manually examined the significances of the gene number differences between the gene orthogroups of spiders and other species by using a Mann‒Whitney *U* test in R.

### Transcription module analysis

We identified orthologs with similar expression patterns using the iterative signature algorithm (isa) implemented in the R package isa2 v0.3.5 [33] with default parameters, following the methods from Zancolli et al. [87]. This algorithm identifies sets of genes that exhibit coherent expression patterns over subsets of samples from a large expression data matrix in an unsupervised manner. It selects genes that are significantly under- or over-expressed in a random seed of samples, and then all samples are scored by the weighted average expression levels across these genes. GO enrichment was performed by KOBAS. We then used REVIGO (http://revigo.irb.hr/) to cluster the overrepresented GO terms and construct the interaction of terms [88].

### Gene evolution analyses

#### Positive selection analyses

Individual sites evolving under positive selection for the ancestral branch of spiders were detected using 1983 orthologous genes in ten species. We estimated the phylogenetic tree in RAxML v8.2.0 (-# 1000) [89] using the concatenated sequences of aligned one-to-one orthologous protein sequences. The divergence times were estimated using the TimeTree database (http://www.timetree.org/) [90]. We employed a mixed-effects maximum likelihood approach by using the MEME framework [91] in Hyphy v2.5.25 (http://www.hyphy.org/) [92] with default parameters. MEME allows the distribution of *ω* to vary from site to site (the fixed effect) and also from branch to branch at a site [91]. Episodic positive selection for each site is shown to be significant using the likelihood ratio test.

#### Relaxed selection analysis

To calculate the strength of natural selection for three spider branches, we used the RELAX framework [93] from Hyphy with default parameters based on the 1983 orthologs across ten species. RELAX asks whether the strength of natural selection has been relaxed or intensified along a specified set of test branches. The results of *K*-value < 1 and *P*-value ≤ 0.05 would suggest significant relaxed selection on test branches, indicating relaxed trends or shifts.

## Supplementary Information

**Additional file 1:** **Table S1.** Genomes for comparative analyses. **Table S2.** Information for all RNA-Seq samples used. **Table S3.** Transcription factors in the filtered gene expression matrix of spider venom glands (average TPM > 0.05). **Table S4.** Differentially upregulated genes in the venom glands of the common house spider. **Table S5.** Module colors for toxin-related coding genes in the WGCNA of the venom glands. **Table S6.** GO functional enrichment results of the core network in the spider venom glands. **Table S7.** GO functional enrichments of highly expressed genes in the different glandular tissues. **Table S8.** The specifically expressed genes in the venom glands and silk glands of the common house spider. **Table S9.** Transcription factor *ASH1* in the common house spider. **Table S10.** Sample weight results for each gene expression module generated from isa2. **Table S11.** Functional annotation of 166 genes in the spider venom gland-specific transcription modules. **Table S12.** GO functional enrichment results of 166 genes in the spider venom gland-specific transcription modules. **Table S13.** Differentially expressed genes in spider venom glands based on the ortholog expression matrix. **Table S14.** Fifteen genes were positively selected on the spider ancestor branch and differentially expressed in spider venom glands.

**Additional file 2:** **SI Text 1.** Repeat sequences and non-coding RNA annotation for the common house spider. **SI Text 2.** Co-expression network associated with venom genes. **Fig. S1.** Chromosome-level genome assembly and annotation of *Parasteatoda tepidariorum*. **Fig. S2.** Repeat sequences and non-coding RNA annotation for *P. tepidariorum*. **Fig. S3.** Weighted gene Co-expression analysis (WGCNA) of the venom glands of *P. tepidariorum*. **Fig. S4.** MDS plots for multiple *P. tepidariorum *tissues based on RNA-Seq data. **Fig. S5.** Ortholog expression patterns across species. **Fig. S6.** Expression tree of all samples based on 1,983 orthologs from ten species, including spiders, scorpions, ticks, mites, centipedes and insects. **Fig. S7.** Module preservation results between common house spider venom glands and other glandular tissues. **Fig. S8.** Observed pairwise semantic similarity (SS) scores and permutated ones among tissues from different species. **Fig. S9.** Similarity comparisons between the common house spider silk glands and other tissues. **Fig. S10.** Similarity index (SI) comparisons among the DUGs of tissues from different species. **Fig. S11.** The orthogroup of the TF *ASH1* across ten species. **Fig. S12.** Weighted gene Co-expression analysis (WGCNA) for multiple tissues from *P. tepidariorum*. **Fig. S13.** Enriched GO terms of the cyan module associated with venom genes. **Fig. S14.** Pie chart for tissue-specifically expressed paralogs of venom gene-associated modules. **Fig. S15.** Heatmap of Spearman correlation coefficients between all 100 transcription modules. **Fig. S16.** Schematic diagram of spiders and their closely related chelicerates. **Fig. S17.** TPM heatmap of some TFs in different tissues of the fruit fly.

## Data Availability

All data generated or analyzed during this study are included in this published article, its supplementary information files, and publicly available repositories. All gene expression matrices, gene orthogroup results, codes, and scripts, as well as new chromosome-level genome assembly, are deposited into the ScienceDB Digital Repository [94]. The genome and transcriptome sequencing data are deposited into the NCBI Sequence Read Archive (SRA) database with BioProject accession PRJNA934108. The Hi-C and transcriptome sequencing data are available under accession numbers SRR23448013–SRR23448014 and SRR23434825–SRR23434891.

## Abbreviations

GO: Gene ontology
DUGs: Differentially upregulated genes
WGCNA: Weighted gene co-expression network analysis
TFs: Transcription factors
TPM: Transcripts per million
PCA: Principal component analysis
SS: Pairwise semantic similarity
SI: Similarity index
isa: Iterative signature algorithm
