# Supplementary material for: Genomic and transcriptomic analyses support a silk gland origin of spider venom glands
Source: BMC Biol. 2023 Apr 13;21:82. doi: 10.1186/s12915-023-01581-7 (PMC10099834; doi:10.1186/s12915-023-01581-7)
Supplement: Supplementary file 2 — Additional file 2: SI Text 1. Repeat sequences and non-coding RNA annotation for the common house spider. SI Text 2. Co-expression network associated with venom genes. Fig. S1. Chromosome-level genome assembly and annotation of Parasteatoda tepidariorum. Fig. S2. Repeat sequences and non-coding RNA annotation for P. tepidariorum. Fig. S3. Weighted gene Co-expression analysis (WGCNA) of the venom glands of P. tepidariorum. Fig. S4. MDS plots for multiple P. tepidariorum tissues based on RNA-Seq data. Fig. S5. Ortholog expression patterns across species. Fig. S6. Expression tree of all samples based on 1,983 orthologs from ten species, including spiders, scorpions, ticks, mites, centipedes and insects. Fig. S7. Module preservation results between common house spider venom glands and other glandular tissues. Fig. S8. Observed pairwise semantic similarity (SS) scores and permutated ones among tissues from different species. Fig. S9. Similarity comparisons between the common house spider silk glands and other tissues. Fig. S10. Similarity index (SI) comparisons among the DUGs of tissues from different species. Fig. S11. The orthogroup of the TF ASH1 across ten species. Fig. S12. Weighted gene Co-expression analysis (WGCNA) for multiple tissues from P. tepidariorum. Fig. S13. Enriched GO terms of the cyan module associated with venom genes. Fig. S14. Pie chart for tissue-specifically expressed paralogs of venom gene-associated modules. Fig. S15. Heatmap of Spearman correlation coefficients between all 100 transcription modules. Fig. S16. Schematic diagram of spiders and their closely related chelicerates. Fig. S17. TPM heatmap of some TFs in different tissues of the fruit fly. [file 12915_2023_1581_MOESM2_ESM.doc]

**Supplementary information text 1–2 and supplementary figure 1–17 for:**

**Genomic and transcriptomic analyses support a silk gland origin of spider venom glands**

Bingyue Zhu^1,2,†^, Pengyu Jin^1,†^, Yiming Zhang^1,2,†^, Yunxiao Shen^1,2^, Wei Wang^3,1^ and Shuqiang Li^1,*^

^1^Key Laboratory of Zoological Systematics and Evolution, Institute of Zoology, Chinese Academy of Sciences, Beijing 100101, China

^2^University of Chinese Academy of Sciences, Beijing 101408, China

^3^Guangxi Normal University, Guilin 541004, China

^†^These authors contributed equally to this work.

***Corresponding author:** E-mail: lisq@ioz.ac.cn (Shuqiang Li).

**Supplementary information text**

**SI Text 1 Repeat sequences and non-coding RNA annotation for the common house spider**

We identified ~489.38 Mb of repetitive sequences for the common house spider (*Parasteatoda tepidariorum*), which encompass 43.27% of the total assembly size (**Fig. S2a**). The great majority of these sequences (39.19%) correspond to transposable elements, many of them (25.48%) without detectable homologs in known databases; DNA transposons are the most abundant type (8.61%), followed by retrotransposons, including long interspersed elements (LINEs, 1.41%), short interspersed elements (SINEs, 3.16%) and long terminal repeats (LTR, 0.52%). Different types of non-coding RNAs were also annotated (**Fig. S2b**), including tRNAs, snRNAs and miRNAs. tRNAs accounted for the most non-coding RNA, with a copy number of 705.

**SI Text 2 Co-expression network associated with venom genes**

To explore the expression patterns of venom genes in the common house spider, we performed weighted gene co-expression network analysis (WGCNA) [23] by utilizing multiple tissues (venom glands, silk glands, ovaries and brains; **Fig. S12a and S12b**). To avoid excessive workload, We only used 5–6 venom gland or silk gland RNA-Seq samples. Low expressed genes were filtered (average TPM < 0.05). We selected a soft threshold of 11 based on results from the “pickSoftThreshold” function in the WGCNA package and obtained an approximate scale-free topology (**Fig. S12b**). A total of 1,088 genes showed a high correlation with venom gland traits and were assigned to the corresponding cyan module (**Fig. S12c and S12d**). Gene enrichment analysis showed that genes in cyan module were mainly related to protein post-translational modification, signal transduction, locomotion and so on (**Fig. S13**). These results further suggested cyan module had a strong interaction with venom genes.


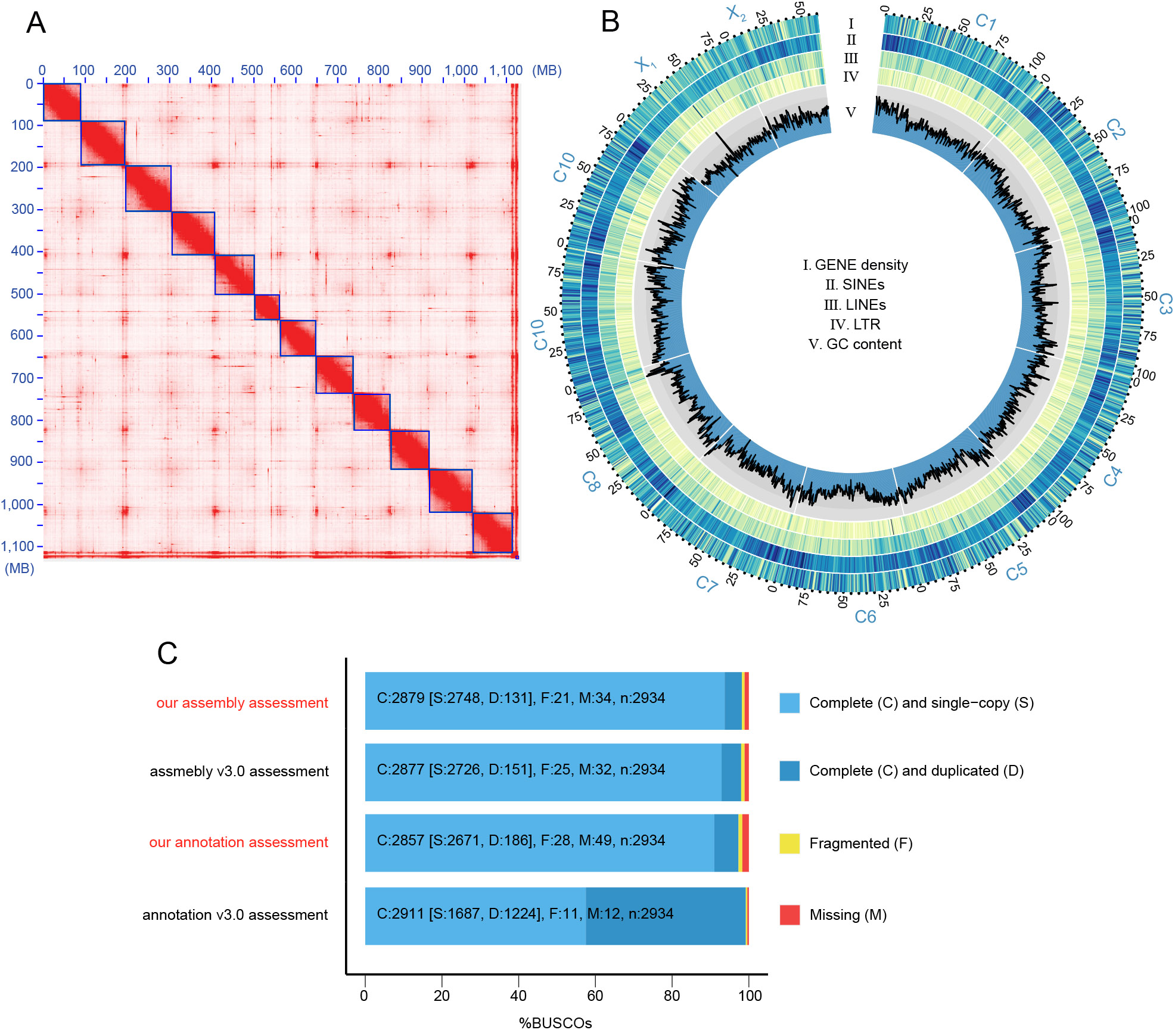


**Fig. S1** Chromosome-level genome assembly and annotation of *Parasteatoda tepidariorum*. **a** Hi-C linkage map. Red blocks indicate the intensity of interaction; blue lines represent the boundaries of chromosomes. Green and blue numbers indicate the sequence length (MB). **b** Circos plot of genome features. The outer ring contains 12 chromosomes. The two smallest chromosomes likely correspond to the X_1_ and X_2_ sex chromosomes. The statistic was based on a 500 kb window. Ⅰ: gene density; Ⅱ−Ⅳ: statistics for several types of repetitive sequences; Ⅴ: GC content. SINEs: short interspersed elements; LINEs: long interspersed elements; LTR: long terminal repeats. **c** The completeness assessment for the common house spider. The completeness of the chromosome-level genome assembly is almost same with version 3.0 (NCBI genome ID: GCA_000365465.3). Our results are highlighted in red.


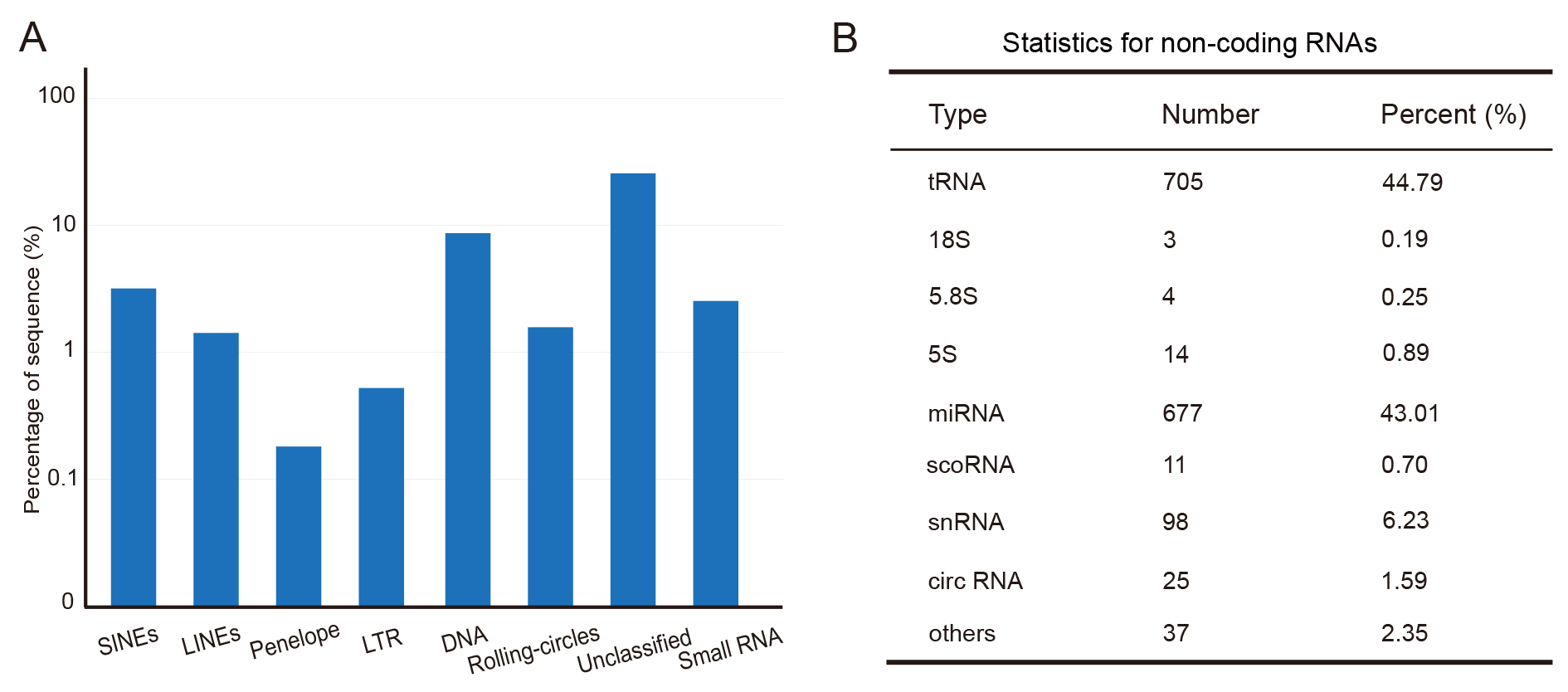


**Fig. S2** Repeat sequences and non-coding RNA annotation for *P. tepidariorum*. **a** Repeat sequences proportion in the genome. Many of them (25.48%) were not detectable homologs in known databases (Unclassified). **b** Non-coding RNA annotation. A total of 1,574 Non-coding RNAs were annotated. tRNAs accounted for the most non-coding RNA, with a copy number of 705.

**
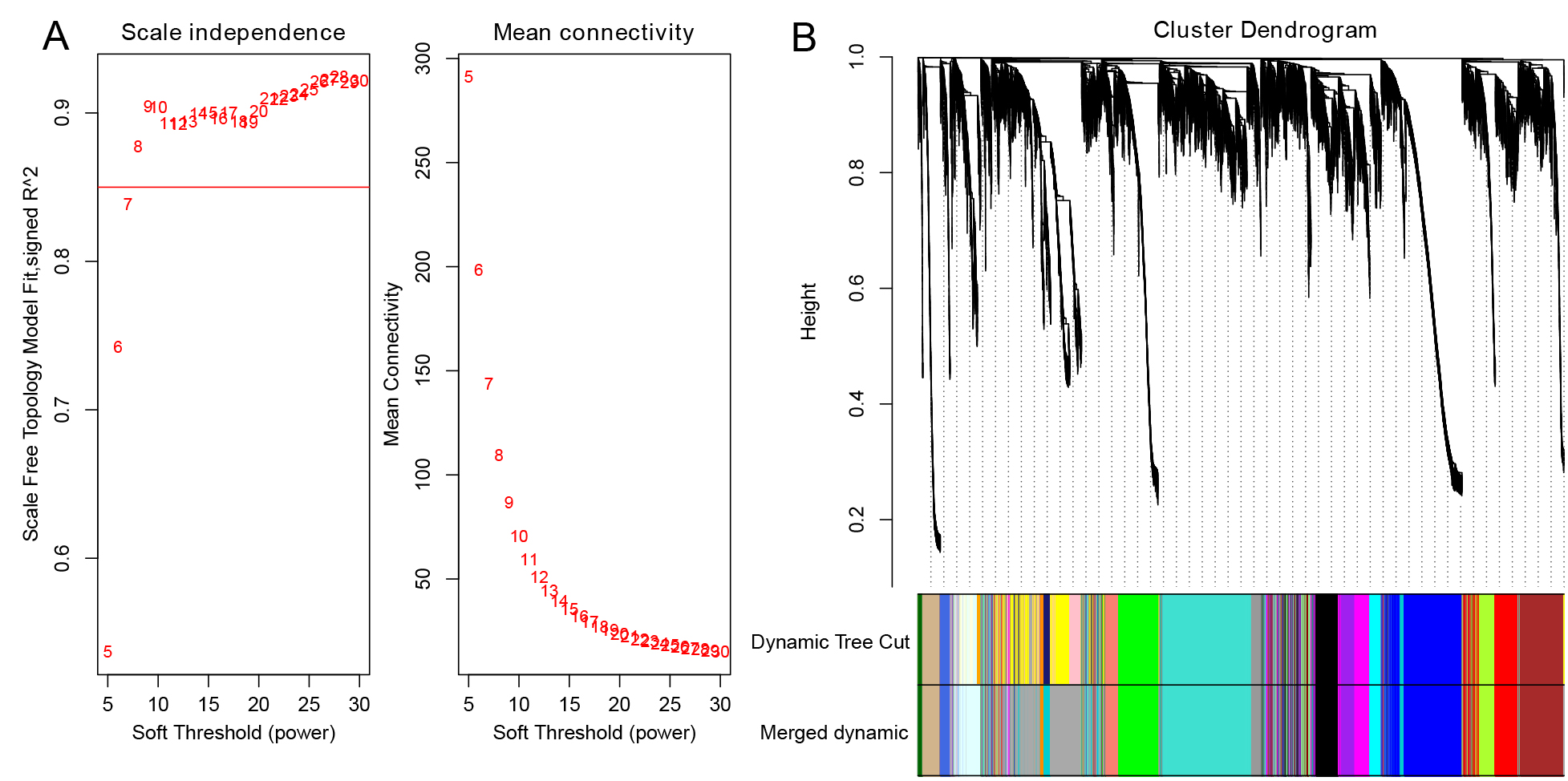
Fig. S3** Weighted gene Co-expression analysis (WGCNA) of the venom glands of *P. tepidariorum*. **a** Analysis of soft-thresholding powers based on scale independence (left) and mean connectivity (right). Soft threshold of 9 was selected. **b** The cluster dendrogram of genes. Modules considered with high similarity were merged.

**
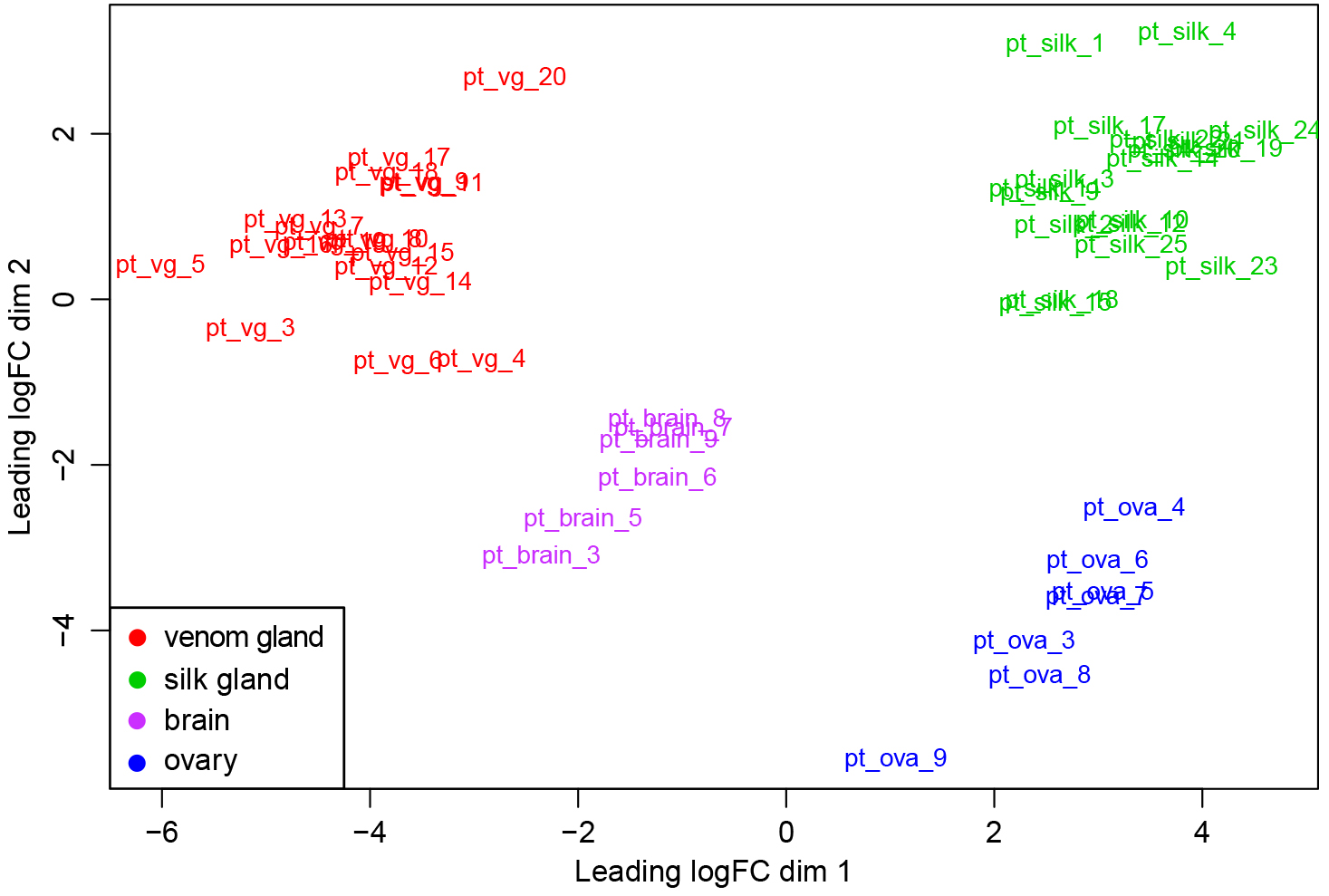
**

**Fig. S4** MDS plots for multiple *P. tepidariorum* tissues based on RNA-Seq data. A total of 50 transcriptome samples were contained in this plot. These samples were used for differential expression analysis.


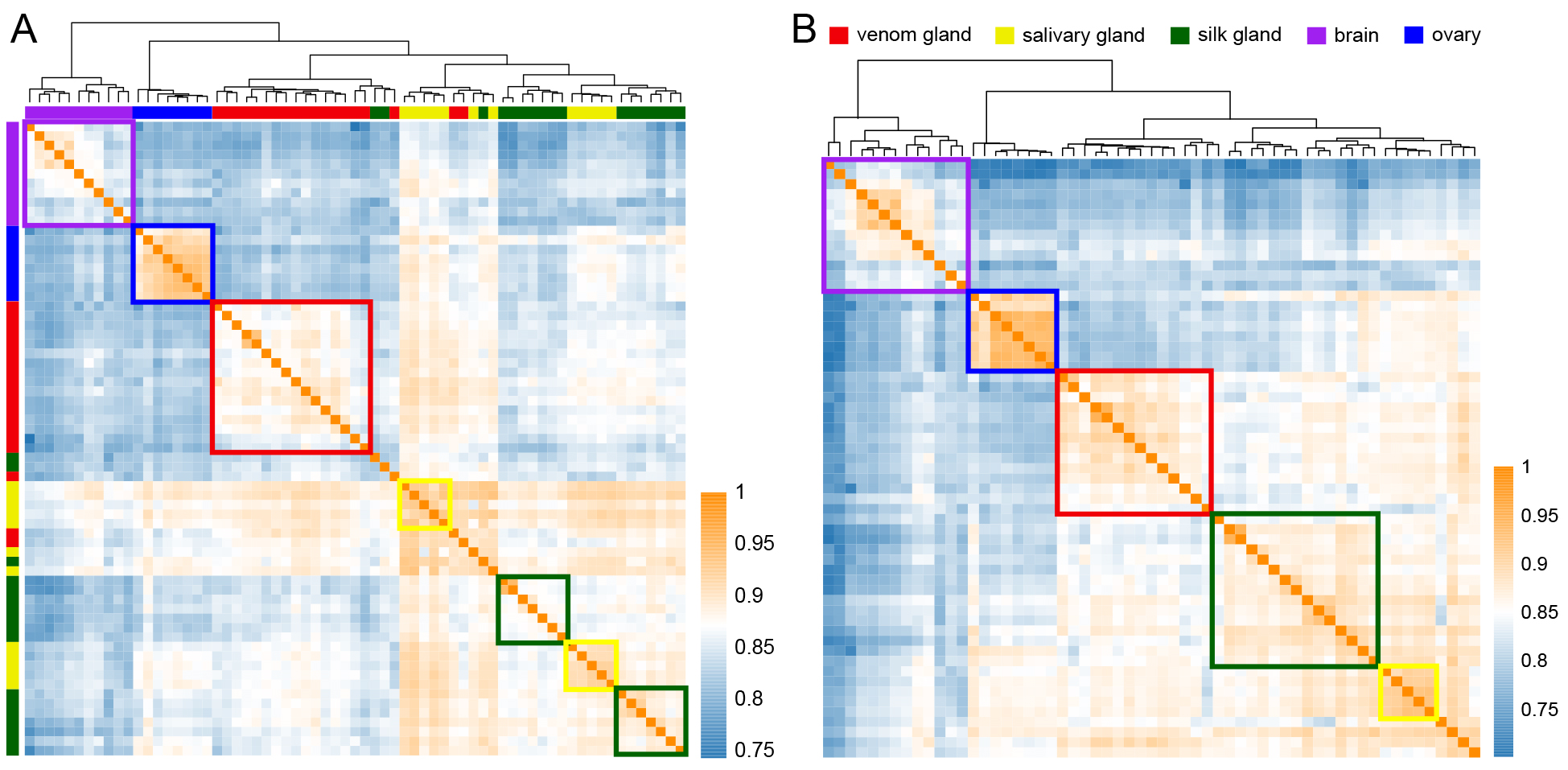


**Fig. S5** Ortholog expression patterns across species. **a** Symmetrical heat map of Spearman’s correlation coefficients between all pairs of samples using 2,388 genes. These orthologs were obtained from six species, including spiders, scorpions, ticks and mites. **b** Symmetrical heat map of Spearman’s correlation coefficients between all pairs of samples using 3,952 orthologs from four species (three spiders and a scorpion).


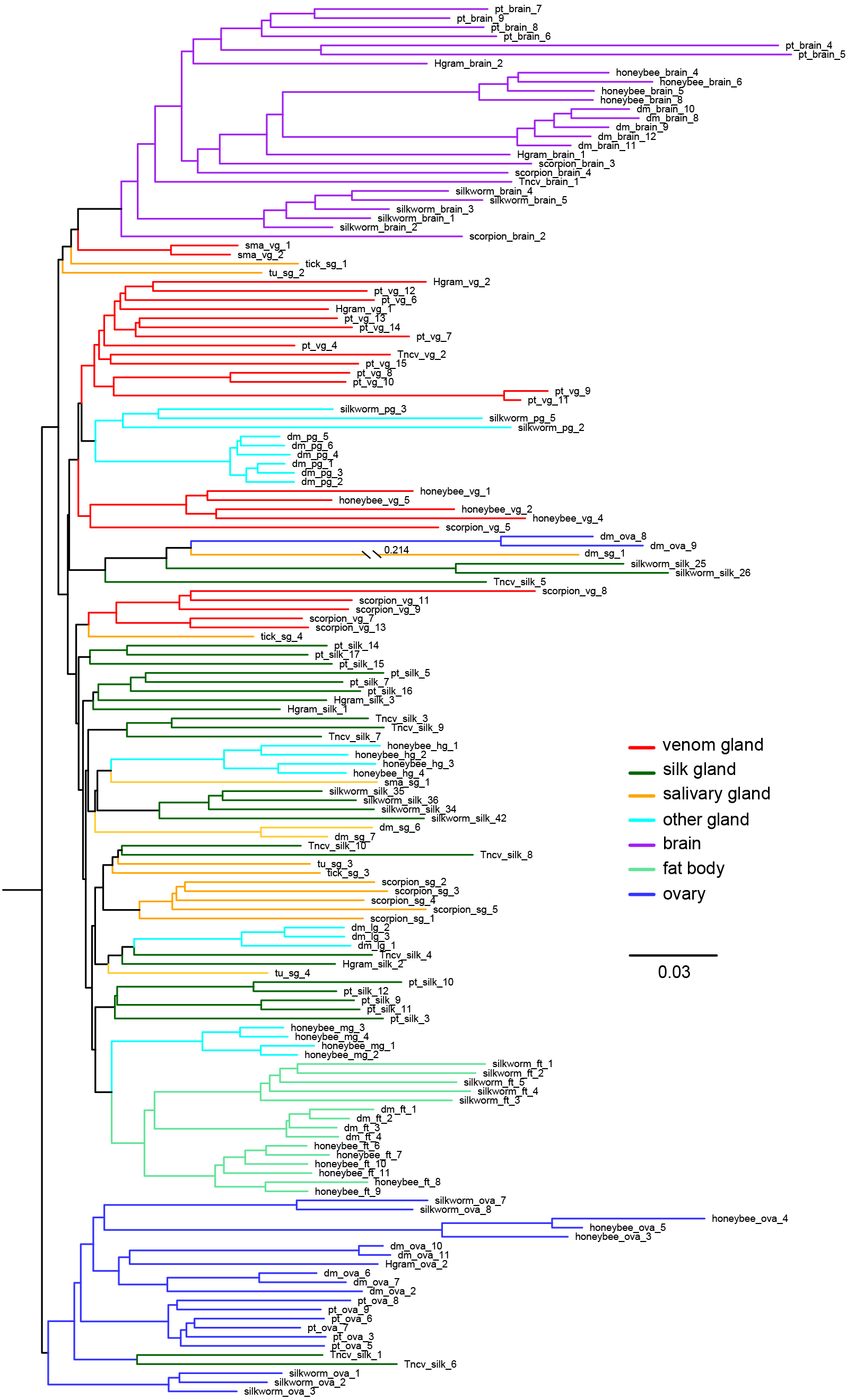


**Fig. S6** Expression tree of all samples based on 1,983 orthologs from ten species, including spiders, scorpions, ticks, mites, centipedes and insects.


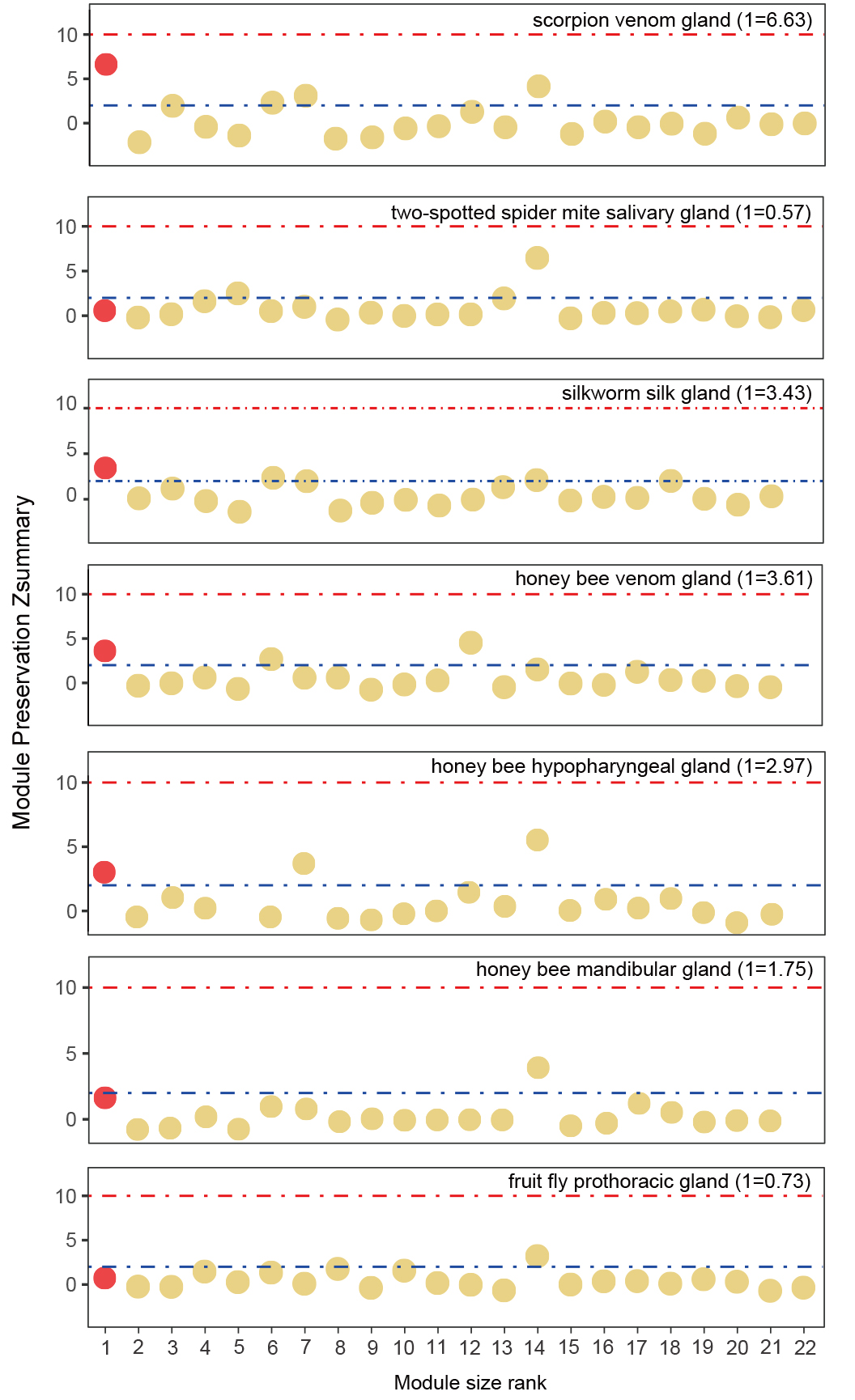


**Fig. S7** Module preservation results between common house spider venom glands and other glandular tissues. Z_summary_ > 10 implies strong preservation; Z_summary_ between 2 and 10 indicates weak to moderate evidence of preservation; if Z_summary_ < 2, there is no evidence that the module is preserved. Red dot (module 1) represents the core network of spider venom glands. The Zsummary value was 6.63 in scorpion venom glands, indicating moderate preservation. The core network has weak preservation in silk gland of silkworm, venom glands of honey bee, and hypopharyngeal glands of honey bee. Other comparison results showed no module preservation.

**
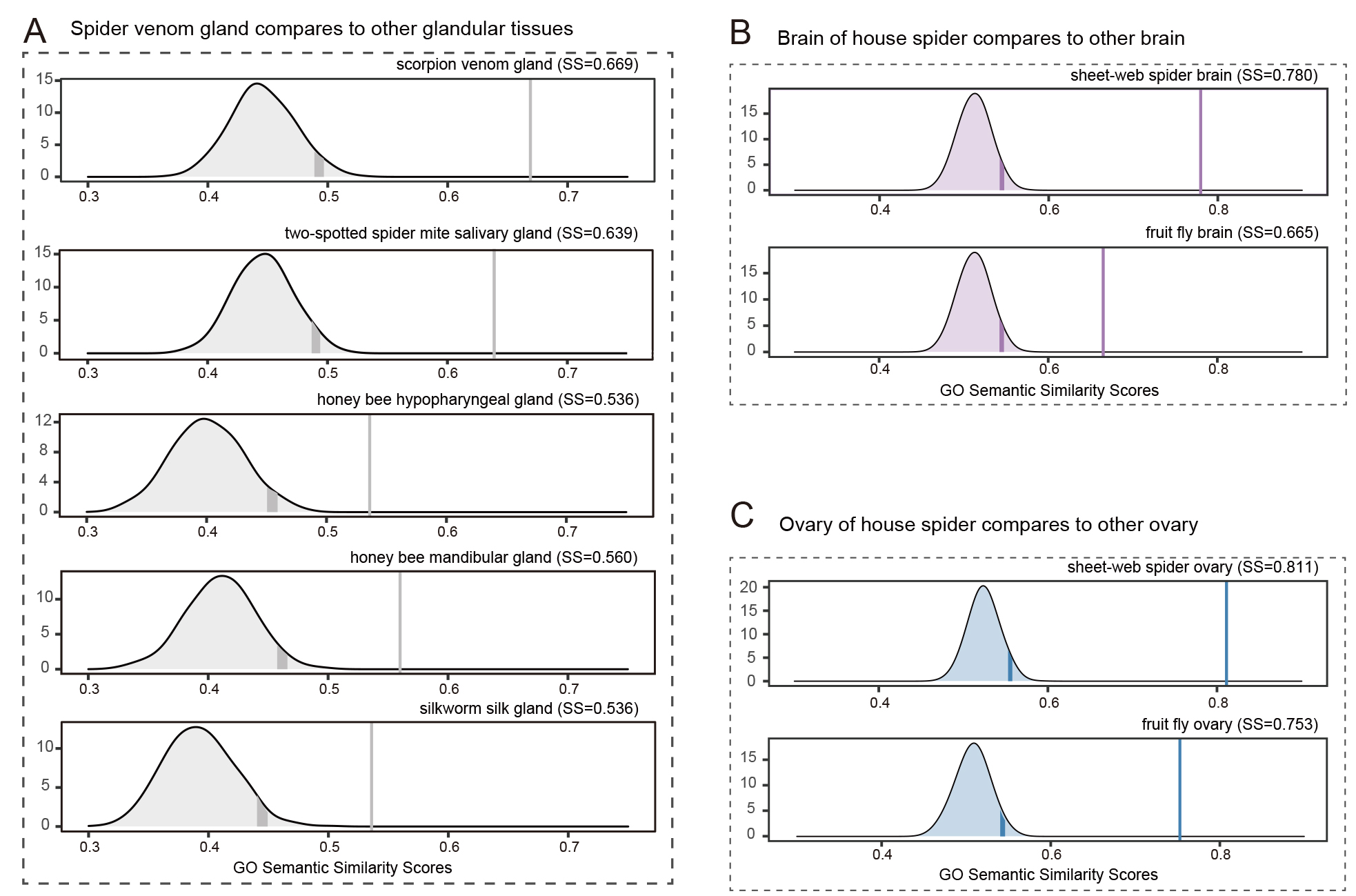
**

**Fig. S8** Observed pairwise semantic similarity (SS) scores and permutated ones among tissues from different species. The vertical lines show the observed pairwise SS values. The shades show 1,000 permutated SS values with 95^th^ and 90^th^ percentiles labeled. **a** SS comparisons between common house spider venom glands and other glandular tissues. SS value of GO enrichments between two spider venom glands was 0.721, signifying the high similarity; the value between the venom and silk gland was 0.672, and the values between venom and salivary glands (scorpion and fruit fly) were 0.569 and 0.548, respectively (**see Fig. 2e in the main text**). These results implied no high similarity between venom and salivary glands. **(b, c)** SS comparisons between the common house spider brain and other brains (b), as well as between the common house spider ovary and other ovaries (c). Our results show a relatively high similarity among the same tissues, suggesting that the small similarity between the spider venom glands and salivary glands of other species is not due to cross-species comparisons. These values were used as the controls in our analysis.


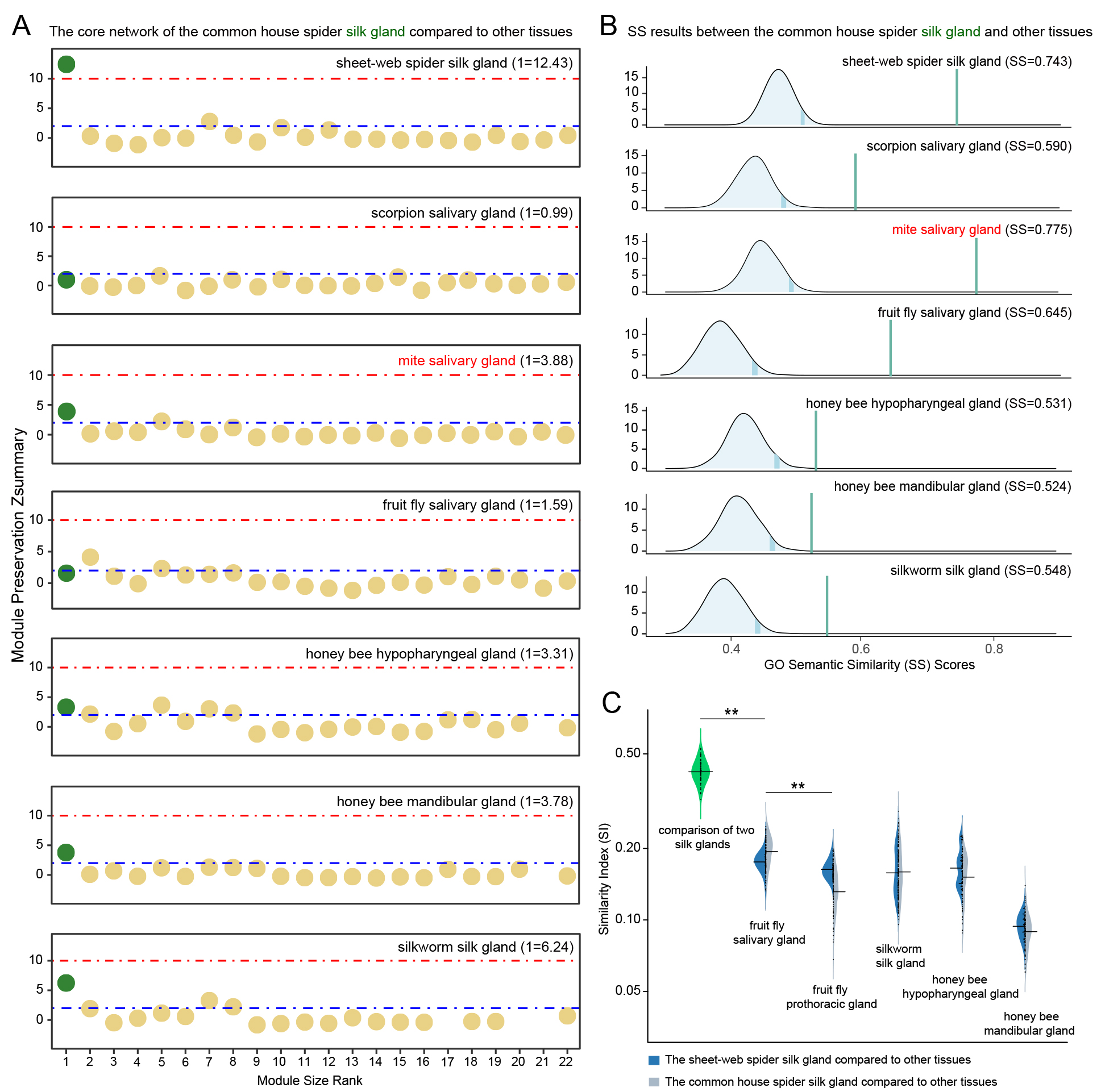


**Fig. S9** Similarity comparisons between the common house spider silk glands and other tissues. The SS value between venom glands and mite salivary glands was not low **(0.639; see Fig. S8a)**, this may be because these mite salivary gland samples also contained other head tissues (e.g., silk glands in mites) [28], and spider mite salivary glands might secrete toxins [29]. **a** Module preservation between the common house spider silk glands and other tissues. In the salivary gland of scorpion and fruit fly, the core network of spider silk gland has no preservation. However, The Z_summary_ value was 3.88 in mite salivary glands, indicating weak preservation. **b** The SS results between the common house spider silk glands and other tissues. The similarity between silk glands and mite salivary glands (0.775) is significantly higher than the value between the silk glands of two spiders (0.743). These high similarities further reflect the abnormality of the mite salivary gland samples. **c** Similarity index (SI) results between DUGs of spider silk glands and other glandular tissues. Blue means the sheet-web spider silk glands compared to other tissues; grey means the common house spider silk gland compared to other tissues. Green represents two spider silk glands compared to each other.


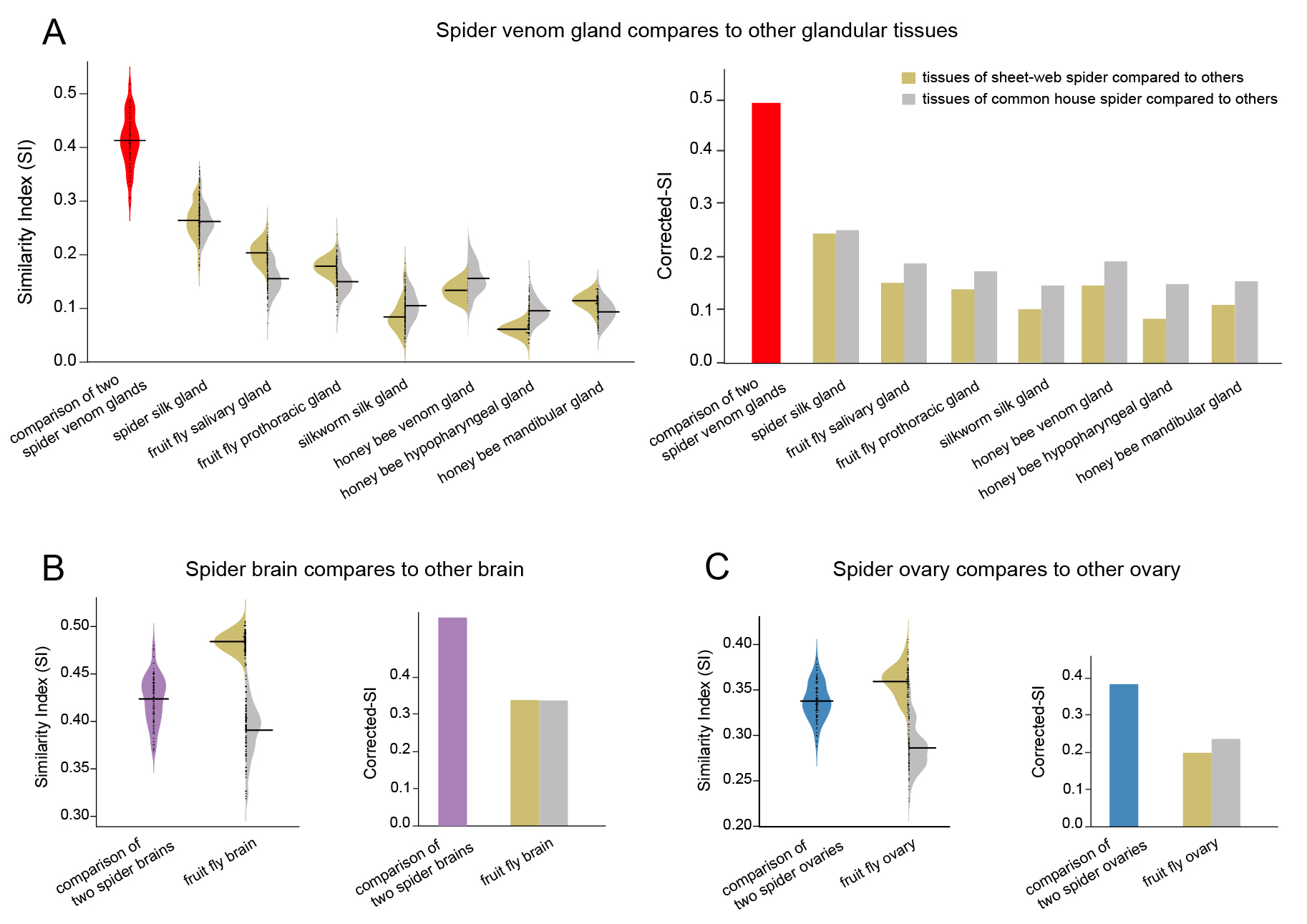


**Fig. S10** Similarity index (SI) comparisons among the DUGs of tissues from different species. This plot corresponded to **Fig. 2g in the main text**. **a** Similarity comparison results between DUGs of spider venom glands and other glandular tissues. **(b, c)** Comparison results between DUGs of spider brain and other brains (b), as well as between spider ovaries and other ovaries (c). The DUGs of the same tissues (from spiders or different species) displayed the relatively high similarities, overcoming differences among taxa, as well as further suggesting cross-species is not the reason for the low similarity between venom glands and salivary glands. Light yellow means tissues of sheet-web spider compared to others; grey means tissues of common house spider compared to others. Red represents two spider venom glands compared to each other; purple indicates two spider brains compared to each other; blue means two spider ovaries compared to each other. The SI results are shown using a bean plot, the corrected-SI results are drawn using a bar plot.


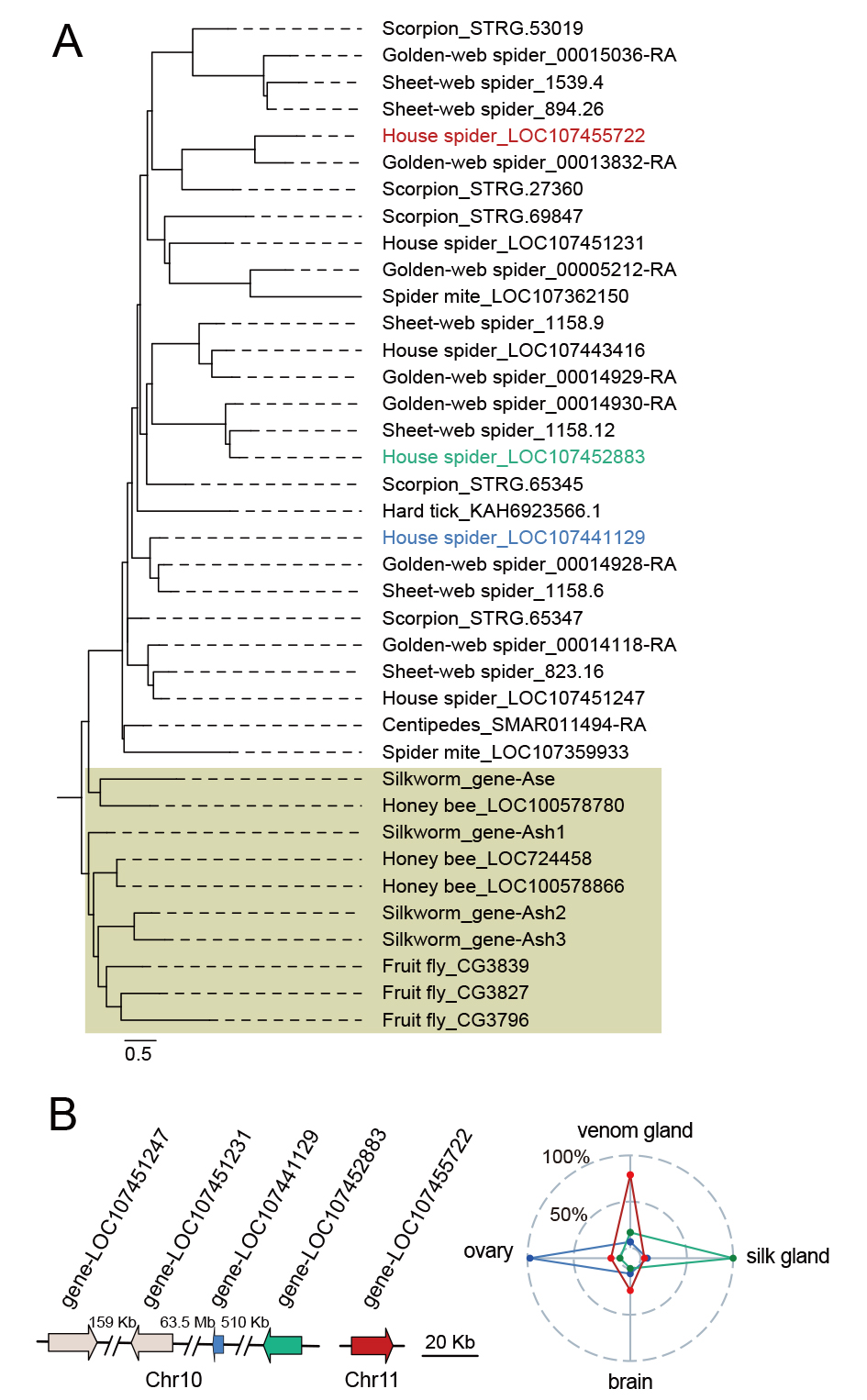


**Fig. S11** The orthogroup of the TF *ASH1* across ten species. **a** The gene tree of TF *ASH1* (orthogroup ID: OG0000298) across ten species is shown as an example. This orthogroup displays ancient gene duplication in arachnids. Genes that are labeled red, green and blue mean the gene is specifically expressed in the venom glands, silk glands and ovaries, respectively. **b** *ASH1* orthogroup in the common house spider. Left: arrayed gene organization; right: expression levels of specifically expressed paralogs in *ASH1* orthogroup. The color meanings are the same to **Fig. S11a**. *ASH1*: achaete-scute homolog 1.


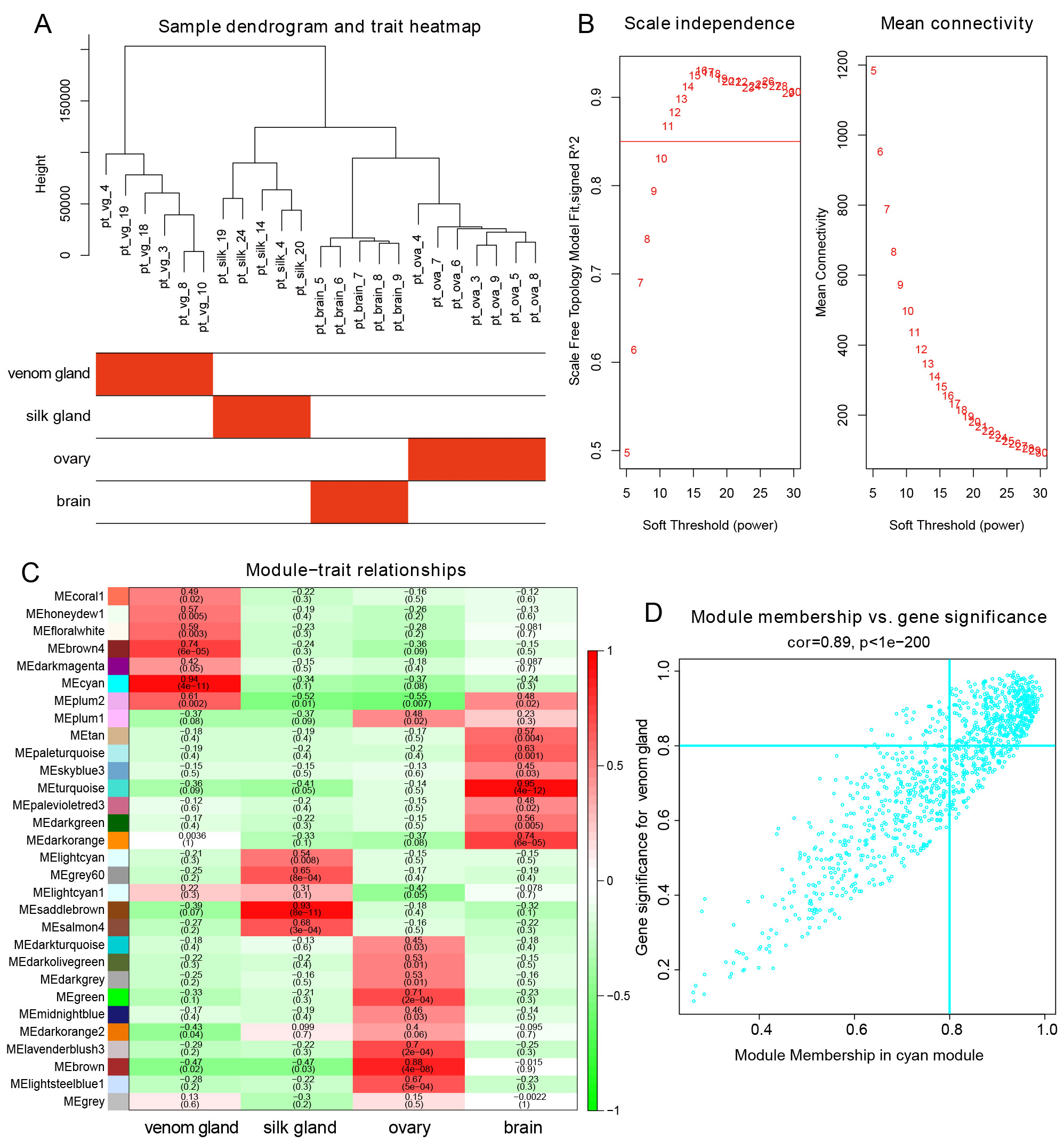


**Fig. S12** Weighted gene Co-expression analysis (WGCNA) for multiple tissues from *P. tepidariorum*. We found the module associated with venom genes from this step. **a** Sample cluster analysis. All of the same tissues cluster together, indicating reasonable biological repetitions. **b** Analysis of soft-thresholding powers based on scale independence (left) and mean connectivity (right). Soft threshold of 11 was selected. **c** Heatmap of correlation between modules and traits. Correlation coefficient along with *P*-value in parenthesis underneath; color-coded according to correlation coefficient. **d** Scatterplot of gene significance (GS) for venom glands versus module membership (MM) in cyan module (venom gland module).


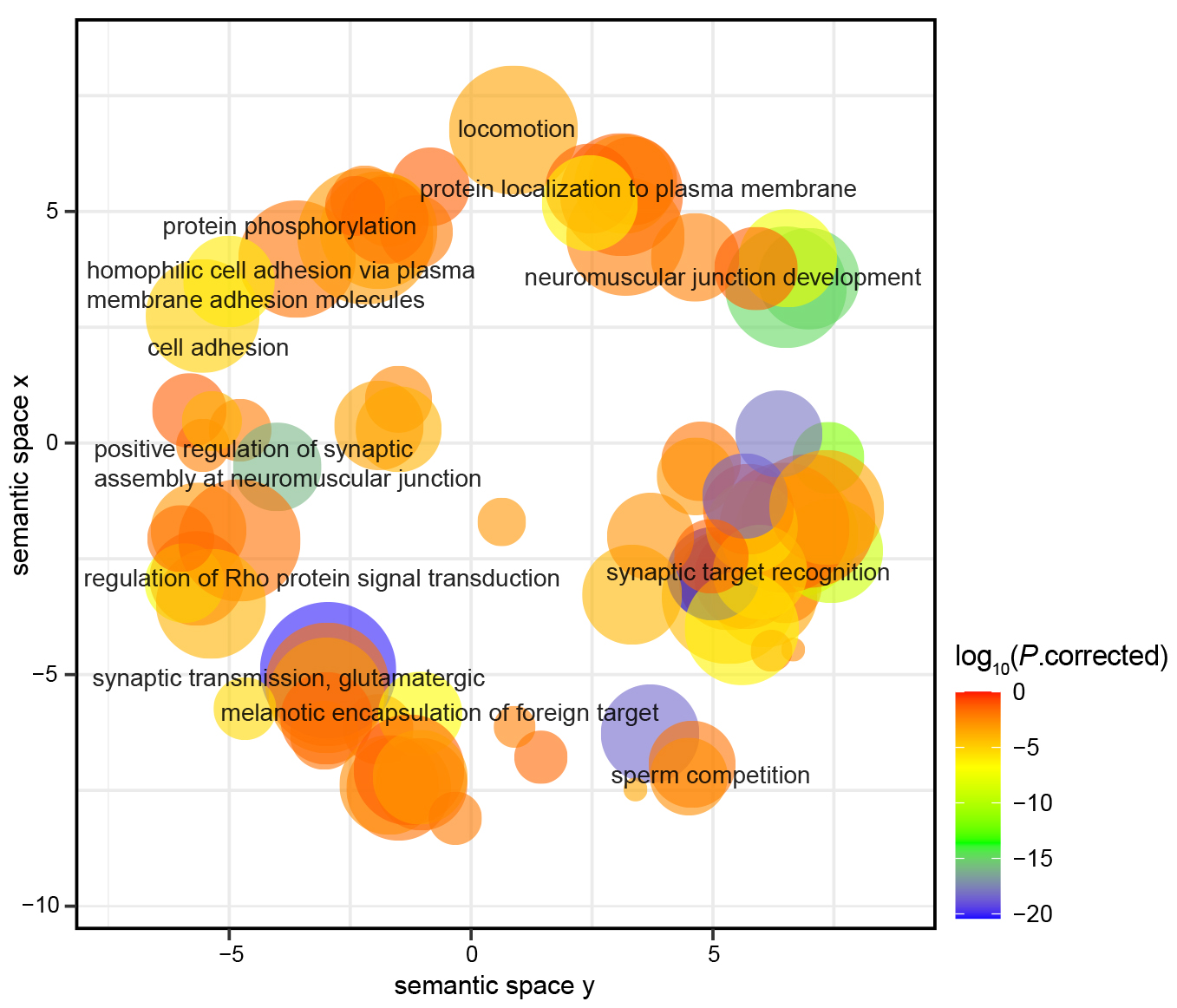


**Fig. S13** Enriched GO terms of the cyan module associated with venom genes. Bubble size represents number of annotations for a certain GO term, and color indicates the corrected enrichment *P*-value on a log_10_ scale.


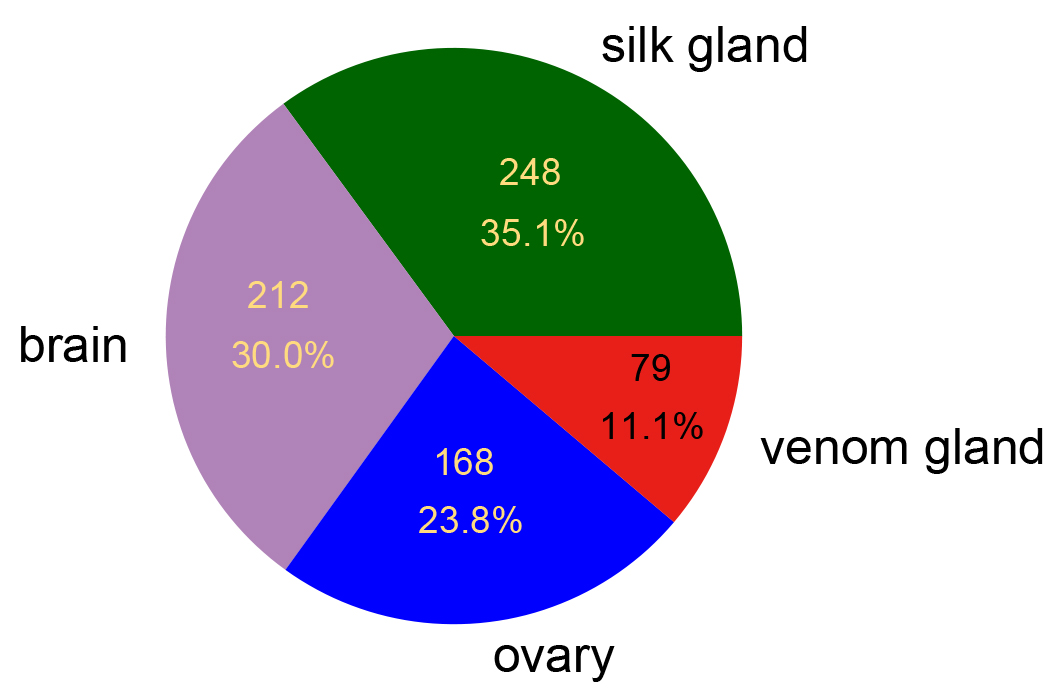


**Fig. S14** Pie chart for tissue-specifically expressed paralogs of venom gene-associated modules. Digits in the pie chart represent the gene number (proportion) of specifically expressed paralogs in four tissues.


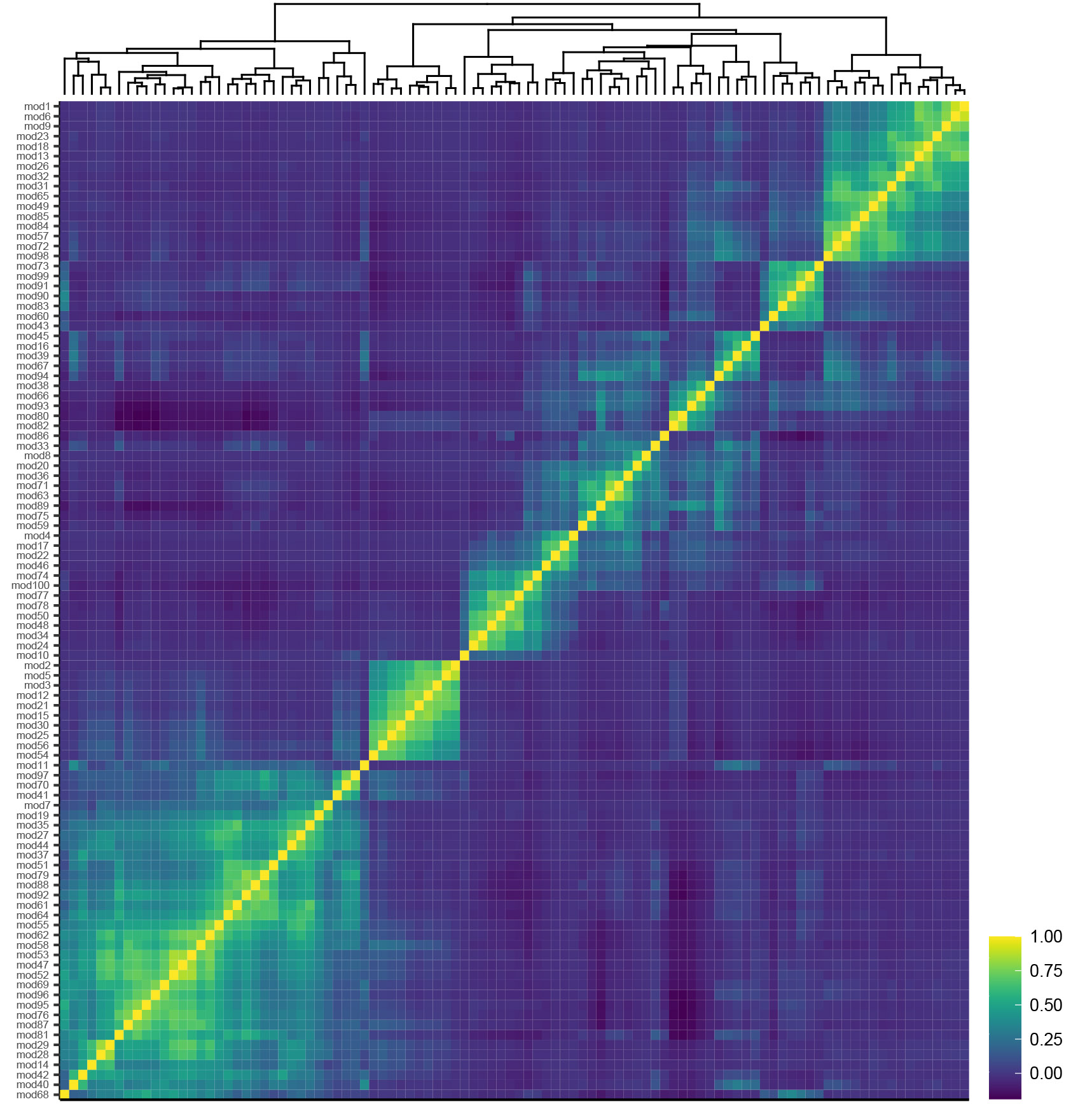


**Fig. S15** Heatmap of Spearman correlation coefficients between all 100 transcription modules. These modules were obtained from the multiple glandular tissues across ten species using isa2 package.


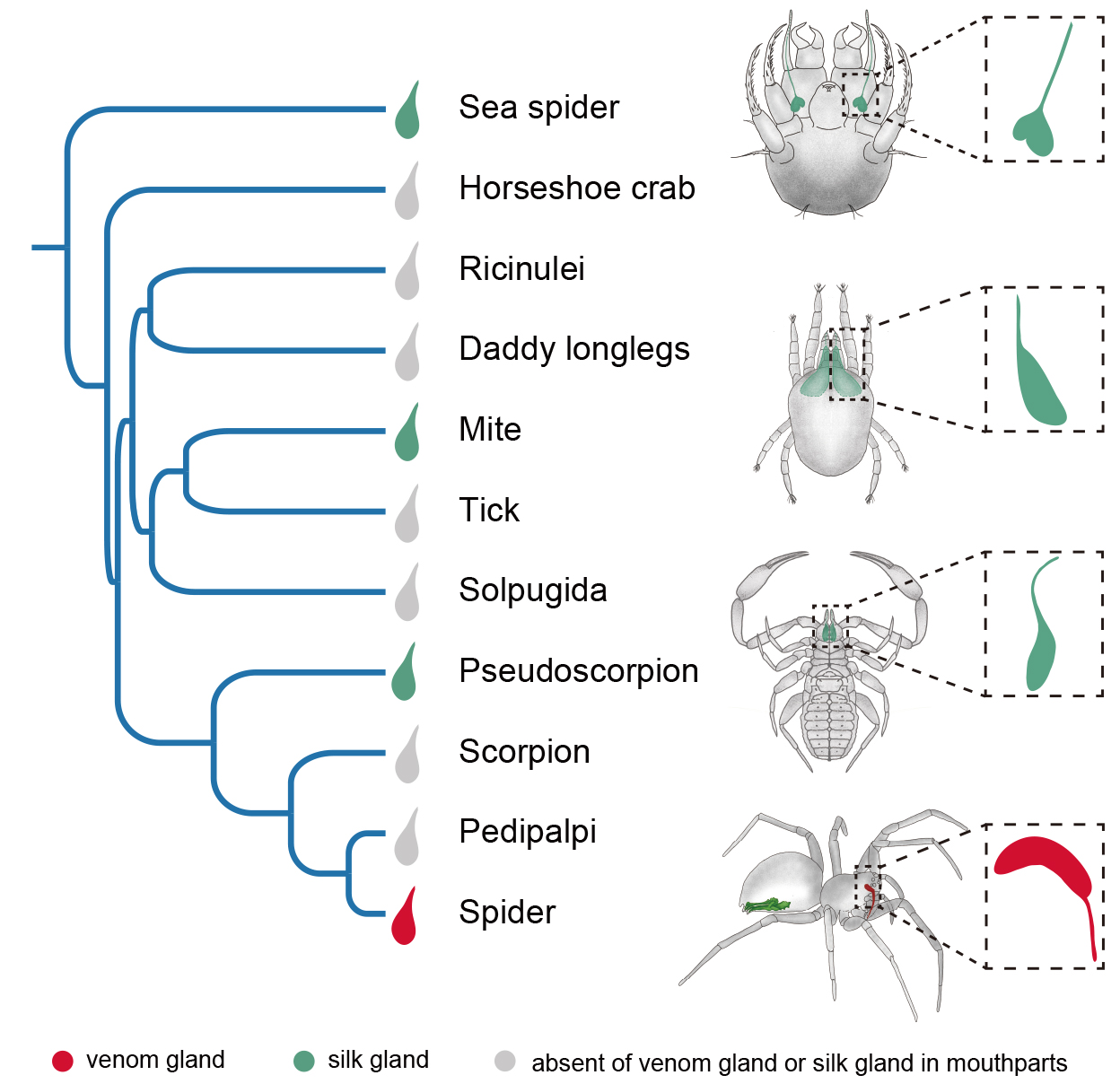


**Fig. S16** Schematic diagram of spiders and their closely related chelicerates. The topology of tree was modified from Lozano-Fernandez et al. [39]. The anatomy plots were modified from previous work [16, 28, 40]. Some pseudoscorpions and larvae of sea spiders have silk-producing glands in their chelicerates; certain mites (Bdellidae and Tetranychidae) have silk glands in their pedipalps. Red: venom gland; green: silk-producing gland; grey: absent of venom gland or silk gland in mouthparts.


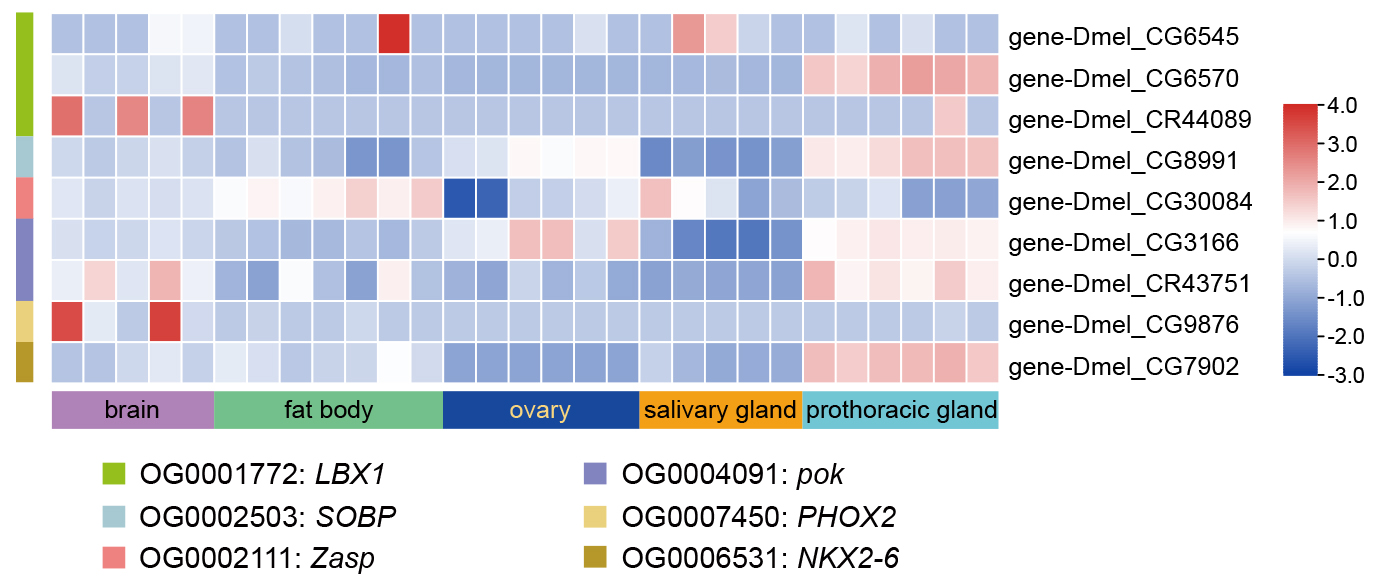


**Fig. S17** TPM heatmap of some TFs in different tissues of the fruit fly. The color blocks below mean the different gene orthogroups and gene names, corresponding to the color bars on the left. *LBX1*: transcription factor LBX1; *SOBP*: sine oculis-binding protein homolog; *Zasp*: PDZ and LIM domain protein Zasp; *pok*: ets DNA-binding protein pokkuri; *PHOX2*: paired mesoderm homeobox protein 2; *NKX2-6*: homeobox protein Nkx-2.6. These TFs were homologous with those TFs presenting strong interactions with toxin gene *CRISP-3* in the common house spider **(see Fig. 3d, e in the main text)**, and showed high expression in the fat body or prothoracic gland of the fruit fly, indicating the formation of specific functional modules and gene recruitment in different tissues across species.
